# Mycobacterial nucleoid-associated protein Lsr2 is required for productive mycobacteriophage infection

**DOI:** 10.1038/s41564-023-01333-x

**Published:** 2023-02-23

**Authors:** Charles L. Dulberger, Carlos A. Guerrero-Bustamante, Siân V. Owen, Sean Wilson, Michael G. Wuo, Rebecca A. Garlena, Lexi A. Serpa, Daniel A. Russell, Junhao Zhu, Ben J. Braunecker, Georgia R. Squyres, Michael Baym, Laura L. Kiessling, Ethan C. Garner, Eric J. Rubin, Graham F. Hatfull

**Affiliations:** 1 Department of Immunology and Infectious Diseases, Harvard T. H. Chan School of Public Health, Boston, MA, USA.; 2 Department of Molecular and Cellular Biology, Harvard University, Cambridge, MA, USA.; 3 Department of Biological Sciences, University of Pittsburgh, Pittsburgh, PA, USA.; 4 Department of Biomedical Informatics and Laboratory of Systems Pharmacology, Harvard Medical School, Boston, MA, USA.; 5 Department of Chemistry, Massachusetts Institute of Technology, Cambridge, MA, USA.; 6 These authors contributed equally: Charles L. Dulberger, Carlos A. Guerrero-Bustamante.

## Abstract

Mycobacteriophages are a diverse group of viruses infecting *Mycobacterium* with substantial therapeutic potential. However, as this potential becomes realized, the molecular details of phage infection and mechanisms of resistance remain ill-defined. Here we use live-cell fluorescence microscopy to visualize the spatiotemporal dynamics of mycobacteriophage infection in single cells and populations, showing that infection is dependent on the host nucleoid-associated Lsr2 protein. Mycobacteriophages preferentially adsorb at *Mycobacterium smegmatis* sites of new cell wall synthesis and following DNA injection, Lsr2 reorganizes away from host replication foci to establish zones of phage DNA replication (ZOPR). Cells lacking Lsr2 proceed through to cell lysis when infected but fail to generate consecutive phage bursts that trigger epidemic spread of phage particles to neighbouring cells. Many mycobacteriophages code for their own Lsr2-related proteins, and although their roles are unknown, they do not rescue the loss of host Lsr2.

Bacteriophages are the most numerous biological entities in the biosphere^1,2^ and possess unparalleled genetic diversity^3^. Host factors needed for phage replication are poorly understood, but mutations both in receptors and intracellular functions can confer phage resistance, and phages must co-evolve in response. This bacterial–phage arms race spans billions of years and dominantly shaped the coevolutionary picture of phages and their hosts^1,2,4^. Together, these factors contribute to viral host range, a key factor influencing phage therapeutic potential^5–10^.

Adsorption to the cell surface is the first step in the phage life cycle. Prevention of adsorption through modification of surface receptors serving as phage entry points is the first line of defence for bacteria, and it is a prevalent target for phage resistance mechanisms^11^. Bacteria thwart phages by acquiring mutations in genes responsible for synthesizing receptors and pathways that secrete extracellular matrix^11^. Resistance can also result from acquiring mutations in host genes that are critical for other stages of the phage life cycle, including those required for phage DNA replication and assembly into mature phage particles^11^.

Mycobacteriophages—phages infecting *Mycobacterium* hosts—are well-studied genomically and are highly diverse. Many are temperate^12^ and enter lysogeny where they replicate passively with the host chromosome^13–16^. Mycobacteriophages have considerable therapeutic potential for *Mycobacterium tuberculosis* and *Mycobacterium abscessus* infections, which are challenging to control due to intrinsic or acquired antibiotic resistance as well as prolonged treatment with harsh antibiotic regimens^8,12,17,18^. The more than 2,000 sequenced mycobacteriophage genomes have been grouped into sequence-related clusters (Clusters A, B and so on), many of which have been divided into subclusters (Subclusters A1, A2 and so on)^19^. Currently, there are 31 clusters (Clusters A–AE) and 7 ‘singletons’, each of which has no close relatives^12,19,20^. All are double-stranded DNA tailed phages, with either siphoviral or myoviral virion morphotypes^12,21^. Their narrow host range among nontuberculous mycobacteria strains limits broad therapeutic potential^22^, but little is known about receptors or other determinants of specificity^23^.

Lsr2 is a nucleoid-associated protein conserved in mycobacteria and actinomycetes encoded by the *lsr2* gene^24^. *M. tuberculosis* Lsr2 is composed of two domains: an N-terminal DNA-binding domain that binds preferentially to AT-rich DNA and a C-terminal oligomerization domain that promotes nucleoprotein filament formation^25,26^. Similar to other bacterial nucleoid-associated proteins, Lsr2 polymerizes around DNA to organize and compact bacterial chromatin^27^ and mediates DNA bridging^28^. Lsr2 is essential for *M. tuberculosis* growth but not for planktonic growth of *M. smegmatis*, although it is required for biofilm formation^29^ and conjugal transfer^30^. Lsr2 is a global gene regulator of cell wall synthesis^26,31–33^ and virulence genes of *M. tuberculosis* and *M. abscessus*^32,34^, and contributes to antibiotic resistance^35^.

Here we show that *Mycobacterium* Lsr2 is required for productive infection by many mycobacteriophages. We observed that phages adsorb specifically to sites of new cell wall synthesis and Lsr2 reorganizes away from chromosomal DNA foci to zones of phage DNA replication (ZOPR). We demonstrate that loss of Lsr2 leads to poor ZOPR establishment, phage resistance and interruption of population-level viral epidemics.

## Results

### Disruption of *M. smegmatis lsr2* confers phage resistance

**Extended Data Fig. 1 |:**
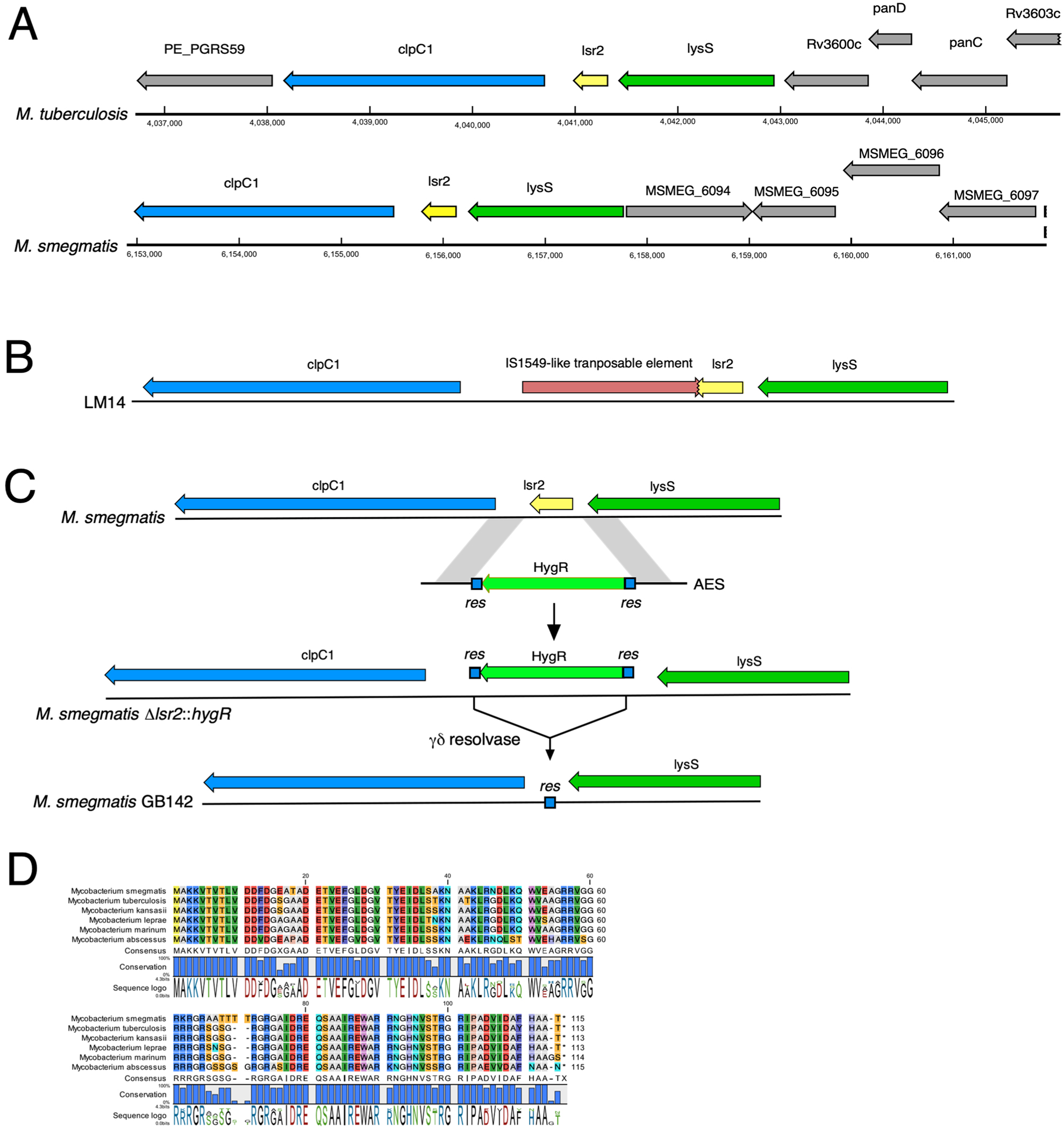
Organization of *lsr*2 locus in mycobacteria. A) Comparison of the *M. smegmatis* and *M. tuberculosis* genomes shows similar arrangements of the clpC1, lsr2 and lysS genes, but variation in the genes flanking those. (B) Position of the IS1549-like transposon inserted into *lsr2* in strain *M. smegmatis* LM14. (C) Two-step construction of *M. smegmatis* GB142 in which a hygromycin cassette flanked by γδ res sites was crossed onto the chromosome by homologous recombination, followed by removal of the HygR gene by resolvase-mediated site-specific recombination. (D) Alignment of Lsr2 proteins from six *Mycobacterium* species.

**Fig. 1 |:**
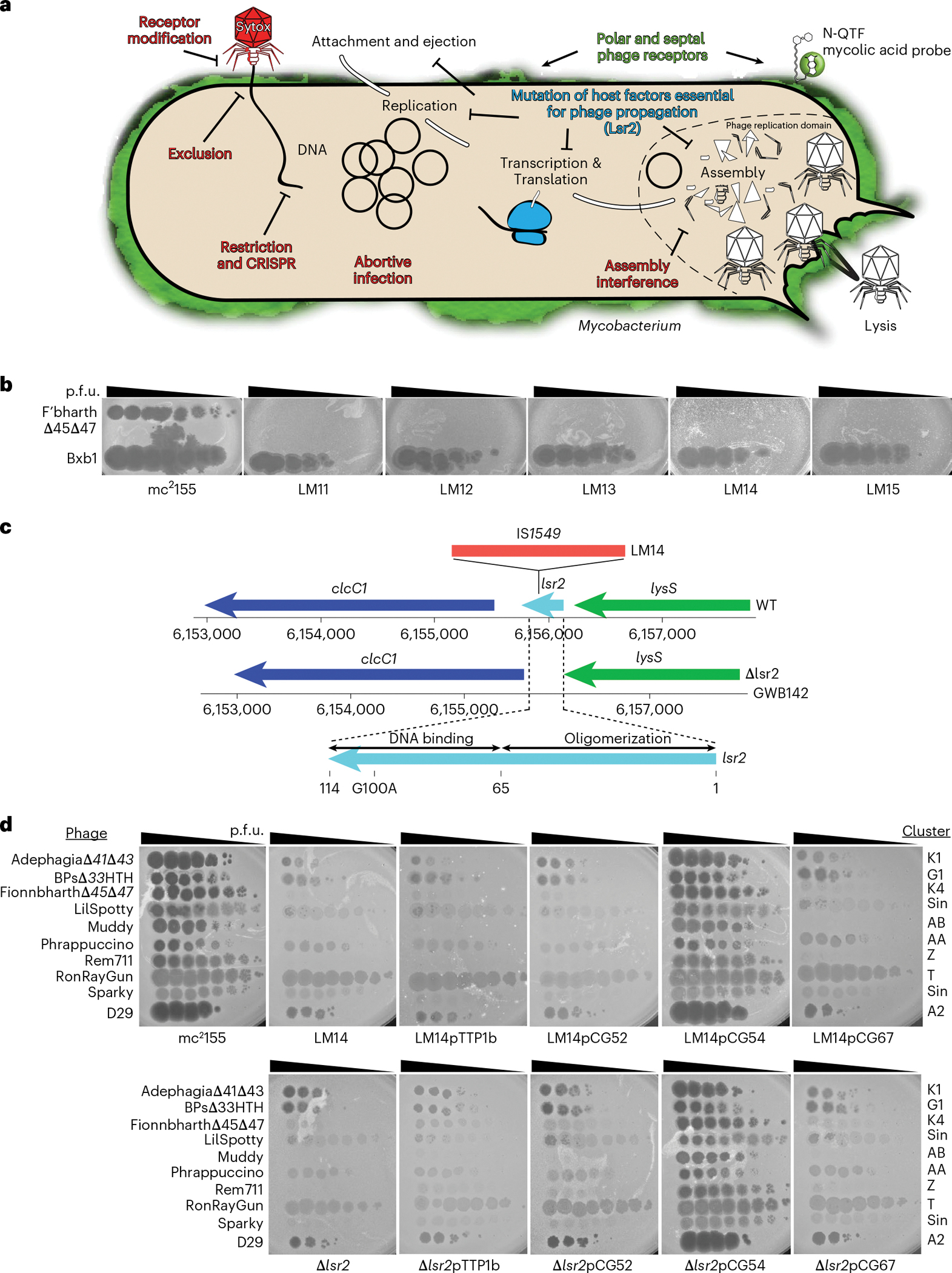
*M. smegmatis* Lsr2 is required for infection of diverse mycobacteriophages. **A**, Schematic of the mycobacteriophage lytic life cycle and resistance mechanisms. Infection begins with adsorption of phage particles to surface bacterial receptors and DNA injection into the host cell. Phage receptors are enriched at the actively growing poles and septa of mycobacterial cells. After DNA injection, the phage hijacks the host replication, transcription and translation machinery to produce and assemble progeny within the phage replication domain (dashed line). The phage expresses lytic enzymes that digest and lyse the host cell envelope, liberating the mature phage particles to initiate new infections. Bacteria resist phage infection via phage defence mechanisms (red text) such as CRISPR, and phage resistance can arise de novo by mutating host bacterial genes (such as *lsr*2) that are essential for phage propagation. **B**, *M. smegmatis* strains LM11–LM15 were isolated as resistant to infection by mycobacteriophage Fionnbharth, using a lytic derivative of the parent temperate phage. Tenfold serial dilutions of phages FionnbharthΔ*45*Δ*47* (in which both repressor and integrase genes are deleted) and Bxb1 were spotted onto lawns of *M. smegmatis* mc^2^155 and LM11–LM15. **C**, Schematic representation of the *M. smegmatis lsr2* locus showing the position of the IS*1549* transposon insertion in the *lsr2* gene (MSMEG_6092) in *M. smegmatis* LM14 and the unmarked *lsr2* deletion mutant GWB142. The bottom shows the domain organization of Lsr2 with amino acid coordinates indicated, together with the location of a G100A substitution in the AT-hook-like DNA binding domain. **D**, Tenfold serial dilutions of a set of genetically diverse mycobacteriophages were spotted onto strains of *M. smegmatis* LM14 and *M. smegmatis* Δ*lsr2* together with their derivatives carrying integrative plasmid vector (pTTP1b), a plasmid with *lsr2* but no promoter (pCG52), a plasmid expressing *lsr2* from a phage BPs promoter (pCG54) or a plasmid derivative of pCG54 carrying a G100A Lsr2 substitution (pCG67); the control strain *M. smegmatis* mc^2^155 on which the phages were propagated is also shown. Phage names are shown at the left and their cluster/subcluster/singleton (sin) designations shown at the right. The variability among independent cultures is shown in Extended Data Fig. 2.

Resistance to phage infection can be mediated by bacterial surface changes resulting in defective binding and DNA injection, or post DNA injection processes that result in either cell death or inhibition of phage replication^36^ (Fig. 1a). Mechanisms of resistance to Cluster K mycobacteriophages and phage Fionnbharth (Subcluster K4) are specifically of interest as these have been proposed for tuberculosis therapy^18^ and the related Cluster K2 phage TM4 is widely used for specialized transduction^37^, diagnostic reporter phages^38^ and transposon delivery^39,40^. We used lytic derivatives of Fionnbharth (FionnbharthΔ*47* or FionnbharthΔ*45*Δ*47*, deleted for repressor or both integrase and repressor, respectively^22^) to isolate resistant mutants of *M. smegmatis* mc^2^155. Five resistant strains were recovered and purified, and designated LM11, LM12, LM13, LM14 and LM15. We confirmed them to be Fionnbharth-resistant (Fig. 1b) but sensitive to unrelated phages such as Bxb1 (Subcluster A1)^41^ (Fig. 1b) and BPs (Subcluster G1)^42^. The five genomes were completely sequenced and compared with the parent strain. LM11 has non-synonymous mutations in several genes including those encoding NAD(P)-dependent alcohol dehydrogenase (MSMEG_4039) and glycogen debranching enzyme GlgX (MSMEG_3186). LM12 and LM13 share the same mutation in a methylmalonyl CoA mutase gene (MSMEG_4881) but have additional mutations elsewhere. Strain LM15 has three mutations resulting in amino acid substitutions: D113G (A3649339G) in MSMEG_3578, E392A (C6970708A) in ABC transporter permease subunit MSMEG_6909 and G93V (C6169539A) in *lsr2* (MSMEG_6092). Strains LM11–LM13 and LM15 were not characterized further. Strain LM14 contains only a single difference from the parent strain: insertion of a resident IS*1549* transposon into the *lsr2* gene MSMEG_6092 (Fig. 1c and Extended Data Fig. 1). The insertion is within the Lsr2 C-terminal DNA-binding domain, with a 13 bp target duplication in codons 93–97. The predicted mutant product is 102 aa long and the C-terminal 17 residues are lost. The Lsr2 protein DNA binding activity is predicted to be lost, although the oligomerization domain may remain functional.

**Extended Data Fig. 2 |:**
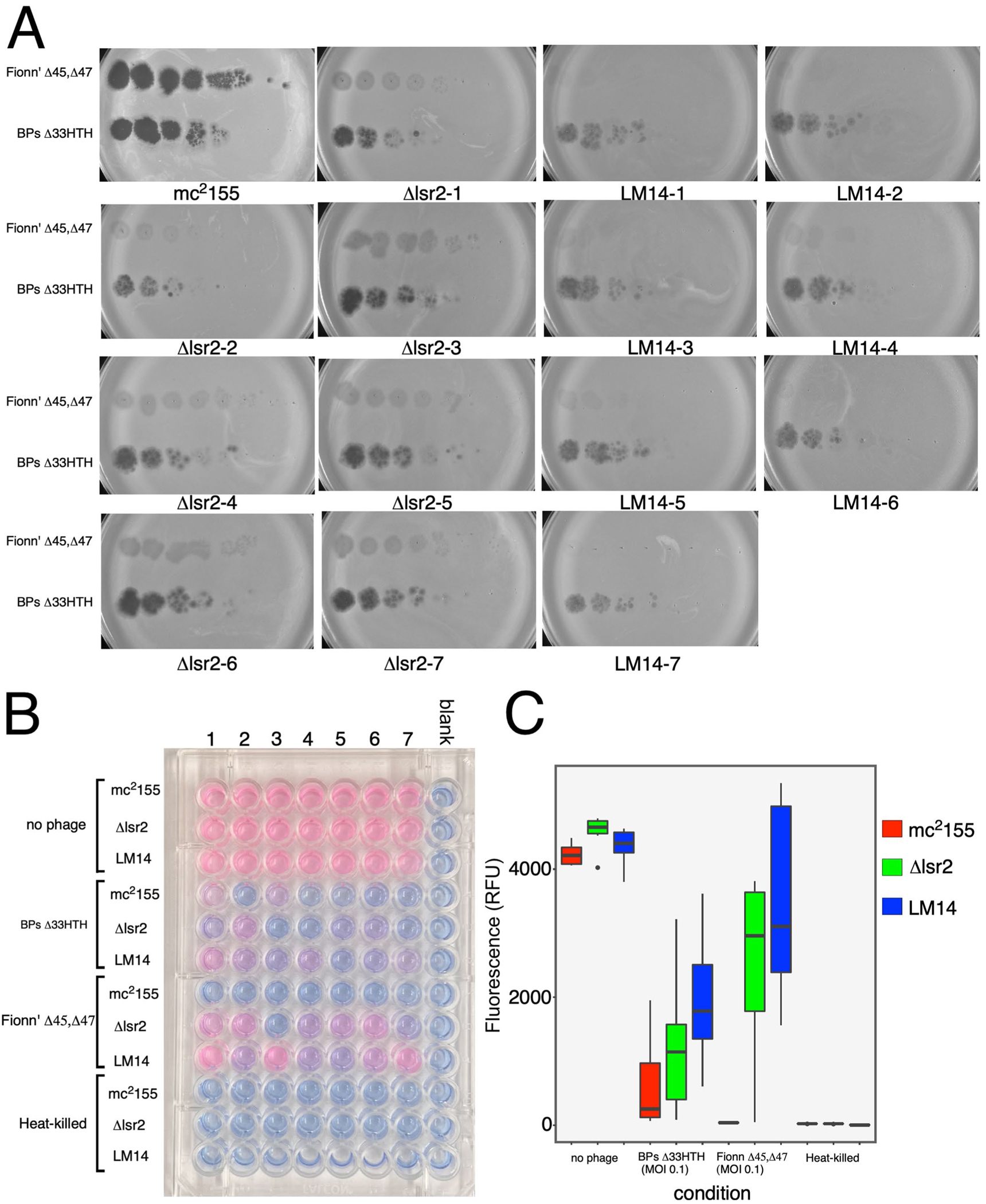
Variability in Fionnbharth infection of LM14 and Δ*lsr2*. (A) Cultures of *M. smegmatis* mc^2^155, *M. smegmatis* Δ*lsr2* and LM14 were grown from single colonies in septuplicate to show phenotypic variation of phage infection of *M. smegmatis* Δ*lsr2* and *M. smegmatis* LM14. Phage lysates were ten-fold serially diluted and spotted onto a single lawn of *M. smegmatis* mc^2^155, and seven for *M. smegmatis* Δ*lsr2* and LM14. (B) An Alamar Blue cell viability assay was performed infecting septuplicates of *M. smegmatis* mc^2^155, *M. smegmatis* Δ*lsr2* and LM14. The top three rows of each set contain uninfected controls, the middle six rows contain wells infected by BPsΔ*33*HTH and FionnbharthΔ45Δ47. The bottom three rows contain heat-killed controls of *M. smegmatis* mc^2^155, *M. smegmatis* Δ*lsr2* and LM14. (C) Box and whisker plots of the quantified relative fluorescence units of the Alamar Blue cell viability assay shown in panel B. Boxes are shaded according to strain as indicated in key. Boxes indicate Interquartile range, dark line inside boxes indicates the median, whiskers indicate minimum and maximum, 1.5 times the value of the interquartile range, outliers are indicated by dots. N = 7 biologically independent cultures.

An *M. smegmatis* strain with an unmarked deletion of *lsr2* (GWB142; Δ*lsr2*) has a phenotype similar to LM14 (Fig. 1d, and Extended Data Figs. 1 and 2). Introduction of integration-proficient vector (pCG54) with a wild-type (WT) copy of *lsr2* driven by a modified P_R_ promoter of phage BPs^43^ complements both strains and restores Fionnbharth infection (Fig. 1d). Recombinant strains carrying vector alone (pTTP1b) or lacking a promoter (pCG52) fail to complement (Fig. 1d). In addition, we tested a plasmid (pCG67) expressing Lsr2 with a G100A substitution in Lsr2^25^ (Fig. 1c*)*, which also fails to complement either strain, showing that the Lsr2 DNA binding domain is required for Fionnbharth infection (Fig. 1d).

**Extended Data Fig. 3 |:**
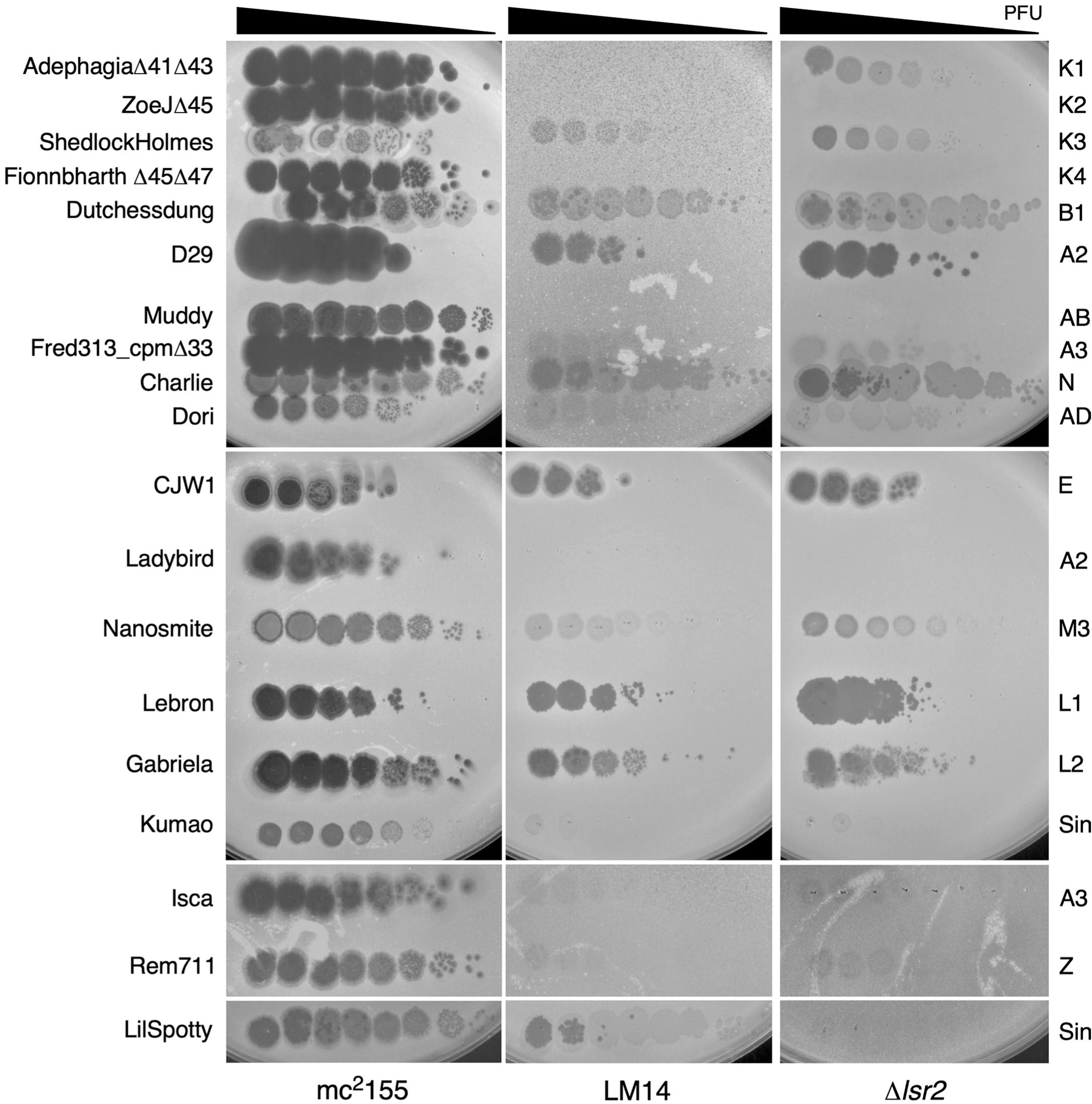
Infection of LM14 and Δ*lsr2* by a diverse panel of phages. Tenfold serial dilutions of a series of genetically diverse mycobacteriophages were spotted onto strains *M. smegmatis* mc^2^155 *M. smegmatis* LM14 and *M. smegmatis* Δ*lsr2*. Phage names are shown at the left, and their cluster/subcluster/singleton (sin) designations are shown at the right.

LM14 and Δ*lsr2* display varying susceptibilities to a diverse panel of mycobacteriophages (Fig. 1d and Extended Data Fig. 3) and only 3 of the 23 phages tested were indifferent to Lsr2 loss (phages Dutchessdung, Charlie and RonRayGun, in Clusters B1, N and T, respectively); all others showed reduction in efficiency of plaquing (for example, Fionnbharth, Muddy), reduced plaque size (for example, D29), increased turbidity (for example, Dori) or a combination of these effects (Fig. 1d and Extended Data Fig. 3). We note that BPsΔ*33*HTH (a lytic derivative of phage BPs) shows a 100-fold reduction in plaquing and increased plaque turbidity relative to *M. smegmatis* mc^2^155 infection. In general, LM14 and Δ*lsr2* strains behaved similarly, with the notable exception of AdephagiaΔ*41*Δ*43*, which forms plaques more efficiently on Δ*lsr2* than on LM14 (Extended Data Fig. 3). For the phages tested, normal infection patterns are restored by complementation (Fig. 1d). Lsr2 thus plays a broad role in mycobacteriophage infection.

### Mycobacteriophages bind at sites of cell wall synthesis

**Fig. 2 |:**
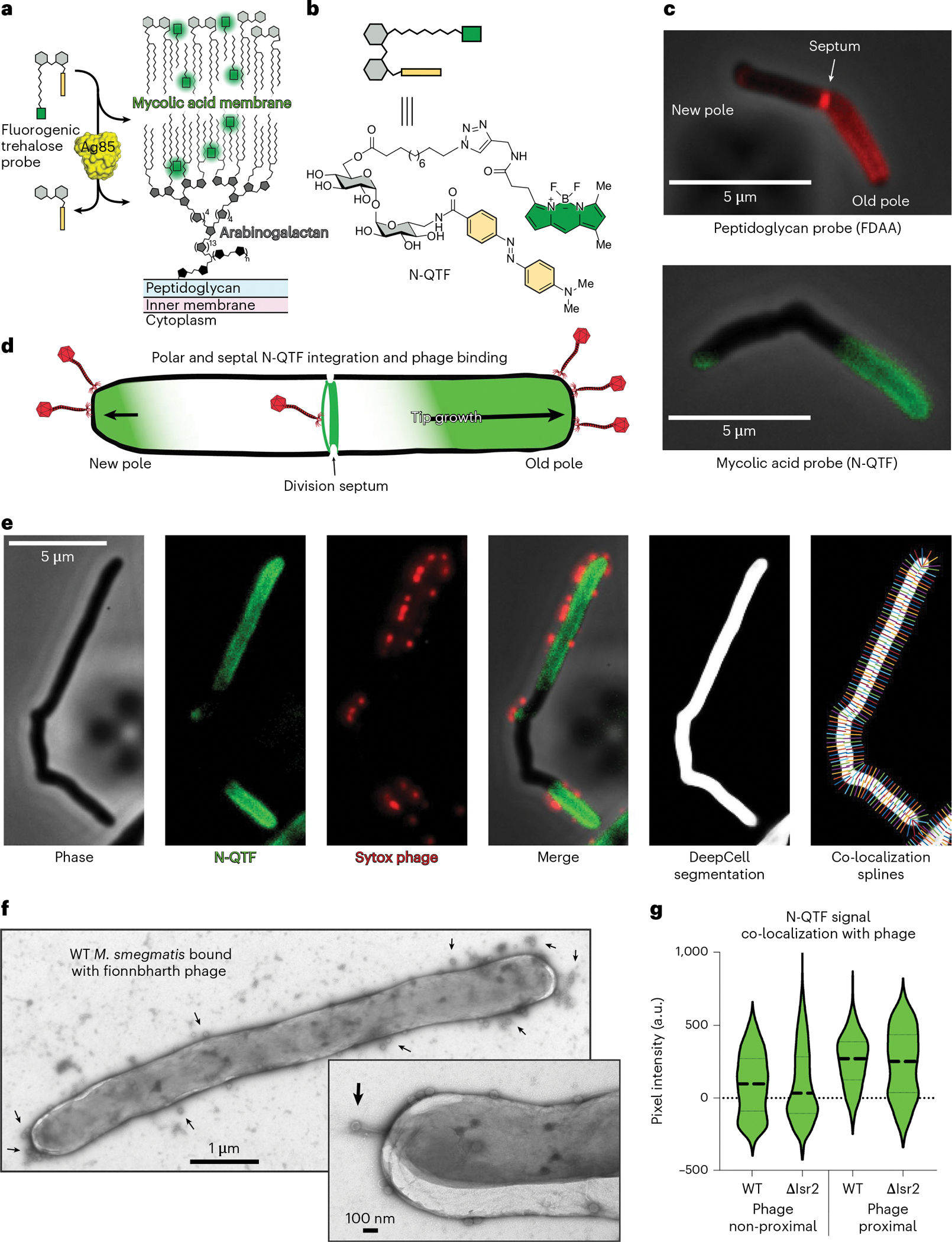
Mycobacteriophage Fionnbharth preferentially binds at sites of cell wall synthesis. **A**, The fluorogenic probe N-QTF is a trehalose monomycolate mimic containing a fluorophore (bodipy, green) and a quencher (yellow). Processing of N-QTF by Ag85 mycolyltransferase removes the quencher and integrates the fluorophore into the mycobacterial outer membrane. These ‘turn-on’ probes allow for monitoring of mycolic acid membrane biosynthesis in real time via imaging and other fluorescence-based readouts. **B**, Chemical structure of N-QTF probe with an amide bond replacing the ester bond linkage between the lipid-fluorophore and the trehalose. N-QTF has improved stability, brightness and membrane-integrating properties. **C**, A comparison of *M. smegmatis* labelling by peptidoglycan- and mycolic acid-integrating probes. The FDAA probe RADA and N-QTF were used at final concentrations of 0.2 mM and 500 nM, respectively. Both label the elongating cell poles and division septa, the sites of active cell wall biogenesis in mycobacteria. The ‘turn-on’ nature of N-QTF allows for the visualization of cell wall synthesis via continuous live-cell labelling and unlike FDAAs, does not require washing out steps. **D**, Schematic depiction of asymmetric polar growth in mycobacteria where the old pole elongates more rapidly than the new pole. This distinctive growth strategy produces a polar and septal labelling pattern seen with cell wall biosynthetic probes and phage adsorption. **E**, Micrographs of a single *M. smegmatis* cell in multiple channels showing the localization of N-QTF incorporated probe (green) and adsorbed SYTOX Orange-stained phages (red) and their colocalization. DeepCell machine learning was used to segment individual cells, and lines perpendicular to the cell surface (splines) facilitated juxtaposition of intramembrane N-QTF pixel intensities with adjacent phage signals. **F**, Negative-stain TEM micrograph of a single *M. smegmatis* cell bound with phages at a MOI of 100. Inset shows a different cell viewed at higher magnification showing individual phages adsorbed to the cell pole. Black arrows highlight bound phages. **G**, Violin plots displaying the quantification of N-QTF-phage colocalization as described in **e**. N-QTF signal intensity is greater at cellular regions proximal to bound phage for both WT and Δ*lsr2* cells. Thick dashed lines denote the median and the thin dashed lines represent upper and lower quartiles.

To determine the role of Lsr2, we developed a set of imaging tools that allowed us to visualize the spatiotemporal dynamics of phage binding to the cell surface and phage replication within infected cells. We used a widefield fluorescence microscope equipped with a CellASIC microfluidic system to collect high resolution time-lapses of the entire Phage life cycle. The N-QTF fluorogenic probe allowed us to continuously track the synthesis of new cell wall material (Fig. 2a–c) and SYTOX Orange-labelled Fionnbharth phage particles^44^ allowed us to resolve single phage particle binding events. With these tools, we observed that phage virions preferentially attach at sites of cell wall synthesis at the poles and septa of mycobacteria^45^ (Fig. 2e–g and Supplementary Video 1). We quantified this co-localization using DeepCell machine learning^46^ to segment individual cells and a custom MATLAB programme using lines drawn perpendicular to the cell surface (splines) to juxtapose the intramembrane N-QTF pixel intensities with the adjacent phage signals (Fig. 2e,g). The N-QTF signal is greatest at cell membrane regions proximal to phage binding events. Similar observations were made with phage Muddy and Adephagia, suggesting that this may be common among mycobacteriophages. Negative-stain transmission electron microscopy (TEM) of phage-infected cells agrees with this observation (Fig. 2f) and shows that Fionnbharth preferentially binds to the growing tips of mycobacteria (Fig. 2f). These data suggest that mycobacteriophage receptors are enriched in polar and septal regions, and that the receptors are intermediates in cell wall biosynthesis that are absent from old or established cell wall material. This may represent a general strategy employed by phages to target actively growing cells that will support lytic replication.

### Role of Lsr2 during mycobacteriophage infection

**Extended Data Fig. 4 |:**
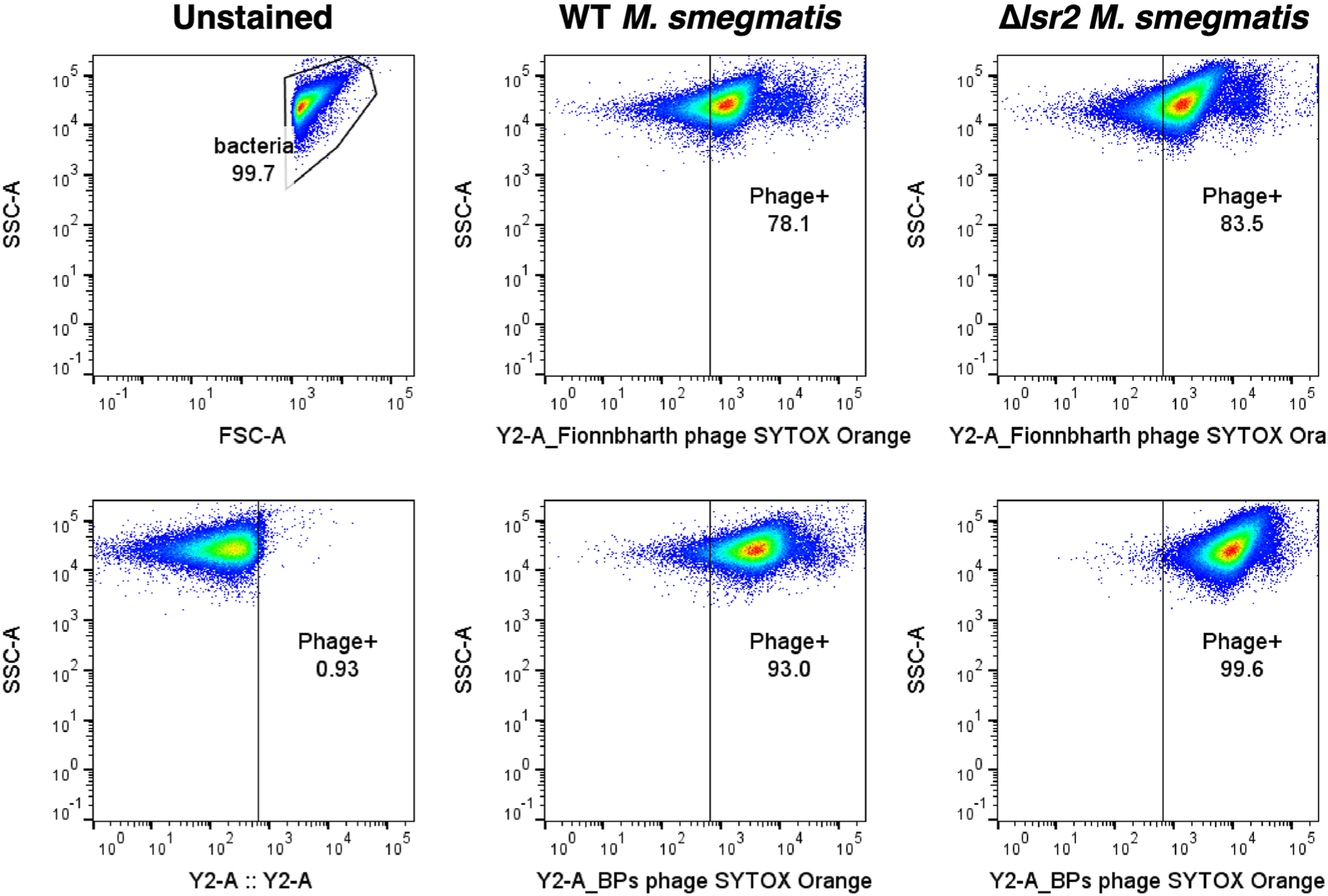
Flow cytometry analysis of phage adsorption. *M. smegmatis* mc^2^155 strains were analyzed by flow cytometry on a MACSQuant (VYB excitation: 488 nm, 561 nm; emission filter: 525/50, 615/20). Bacterial cells were gated on SSC-A / FSC-A and an unstained sample was used as a negative control to draw positive gates for N-QTF and SYTOX Orange fluorochromes. >50,000 events were recorded. Data was analyzed in FlowJo.

**Extended Data Fig. 5 |:**
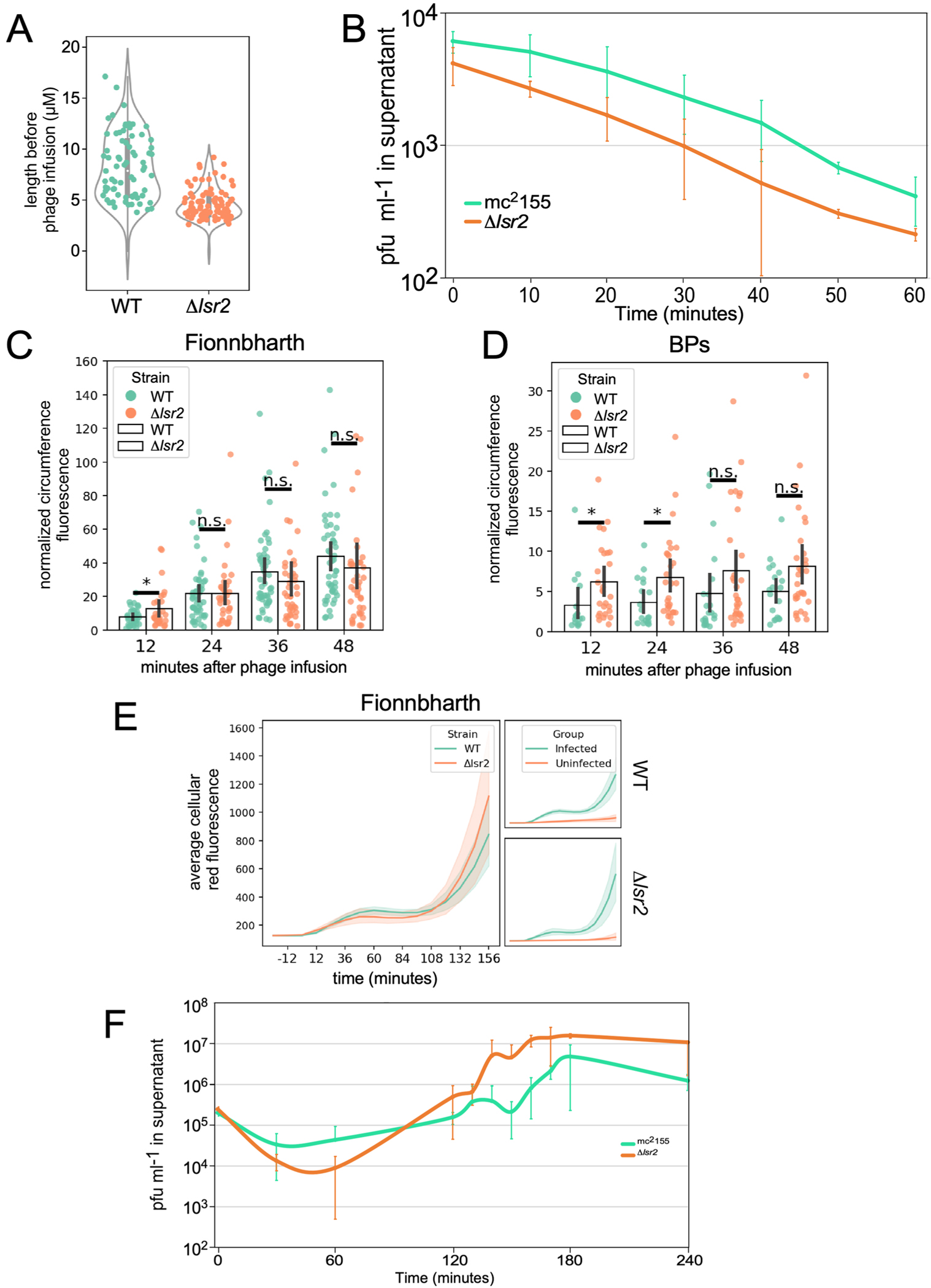
Phage infection of *M. smegmatis* mc^2^155 and *M. smegmatis* mc^2^155 Δ*lsr2*. (**A**) Quantification of cell length from images of *M. smegmatis* mc^2^155 (N = 76) and *M. smegmatis* mc^2^155Δ*lsr2* (N = 87). Violin plots of cell lengths prior to phage infusion are denoted in grey. Green and orange dots indicate single cell measurements of mc^2^155 and mc^2^155Δ*lsr2*, respectively. (**B**) Adsorption of FionnbharthΔ*45*Δ*47* to mc^2^155 (green) and mc^2^155Δ*lsr2* (orange). Cells and phage were mixed at low MOI (0.0001) at indicated times, aliquots were removed, cells were pelleted by centrifugation and the phage titre determined by plaquing on mc^2^155; error bars indicate standard deviation for each data point. N = 3 biological replicates. (**C** and **D**) Quantification of phage adsorption (**C**, FionnbharthΔ*45*Δ*47*; **D**, BPs) to *M. smegmatis* mc^2^155 and *M. smegmatis* mc^2^155Δ*lsr2* from time-lapse imaging. Image segmentation and analysis are performed using bespoke python scripts. Normalized circumference fluorescence of phage absorption was estimated by normalizing the summed intensity of red fluorescence peaks along the cell circumference to the total length of the cell circumference. Error bars denote the standard deviation of normalized circumference intensity profiles. For BPs infection, phage absorption profiles are obtained from 21 and 31 successfully tracked single cells of mc^2^155 and mc^2^155Δ*lsr2*, respectively. For Fionnbharth infection, the corresponding cell counts were 49 and 41. Statistical analysis was performed using the two-tailed Student’s t-test, and a single asterisk indicates p < 0.05. (**E**) Quantification of mCherry expression following infection of *M. smegmatis* mc^2^155 (N = 49) and *M. smegmatis* mc^2^155 Δ*lsr2* cells (N = 41) with Fionnbharth mCherry reporter phages; 2 and 9 uninfected cells of *M. smegmatis* mc^2^155 or *M. smegmatis* mc^2^155Δ*lsr2* are identified in the same imaging fields and their mCherry fluorescence profiles are plotted on the right panels. Shaded areas denote the standard deviation of cellular mCherry fluorescence. (**F**) Phage production following infection of *M. smegmatis* mc^2^155 (green) and *M. smegmatis* mc^2^155Δ*lsr2* (orange) by FionnbharthΔ*45*Δ*47*. Cells were infected at low MOI (0.001) at indicated times, aliquots were removed, cells were pelleted by centrifugation and the phage titer determined by plaquing on mc^2^155; error bars indicate standard deviation for each data point. N = 3 biological replicates.

**Fig. 3 |:**
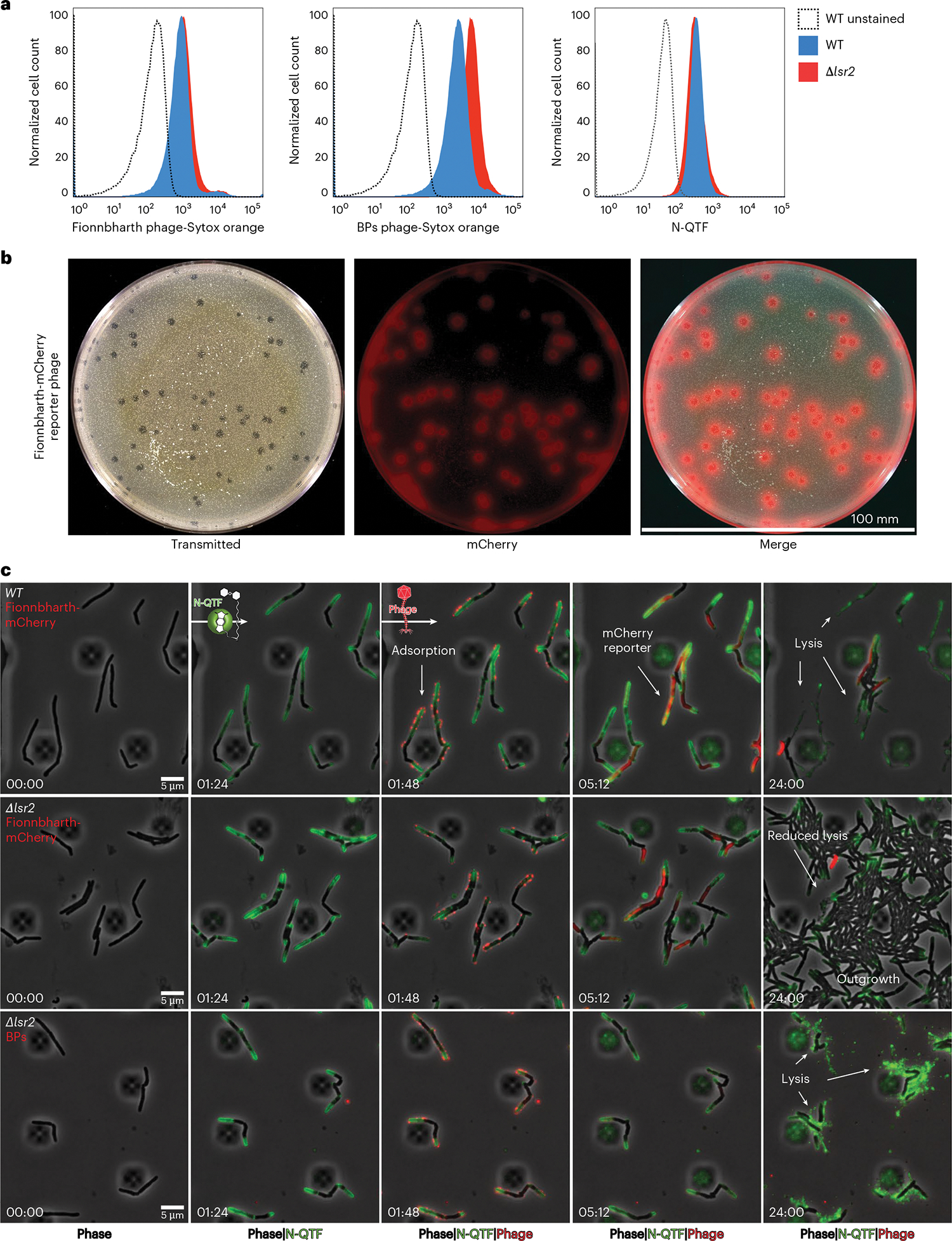
*lsr2* deletion does not inhibit adsorption and protects via a post-injection mechanism. **A**, Flow cytometry data plotted as histograms showing the population-level fluorescent signals for *M. smegmatis* cells labelled with SYTOX Orange-stained mycobacteriophages at a MOI of 100 (Fionnbharth on the left, BPs in the middle) and N-QTF at a concentration of 500 nM. **B**, A plaque assay for the Fionnbharth-mCherry reporter phage. Images depict a 100 mm agarose plate containing a lawn of WT *M. smegmatis* cells infected with 100 phage particles. The plate and fluorescent plaques are shown in the transmitted light channel, mCherry and merged. **C**, Time-lapse of WT or *Δlsr2 M. smegmatis* cells grown and infected with fluorescent phages in a CellASIC microfluidic device. Cells are continuously labelled with N-QTF to mark sites of cell wall synthesis and infected via a 1 h pulse of SYTOX Orange-labelled phage particles at 10^7^ p.f.u. ml^−1^. The Fionnbharth-mCherry reporter signal turns on when proteins are expressed from the phage DNA. White arrows highlight important events in the infection life cycle including adsorption, mCherry reporter signal and lysis vs outgrowth. Wild-type cells are susceptible to Fionnbharth and BPs phages while Δ*lsr2* cells are susceptible to BPs but resistant to Fionnbharth resulting in cell outgrowth.

To determine whether Lsr2 is required for Fionnbharth adsorption, we infected *M. smegmatis* with SYTOX Orange-labelled phages at a multiplicity of infection (MOI) of 10 and analysed cells via flow cytometry (Fig. 3a and Extended Data Fig. 4). Fionnbharth adsorption is qualitatively and quantitatively similar for both WT and Δ*lsr2* cell types, suggesting that Lsr2 is not required for Fionnbharth binding (Extended Data Fig. 5). We similarly tested the binding of SYTOX Orange-labelled phage BPs, for which infection is only mildly reduced by loss of *lsr2* (Fig. 1d). BPs similarly adsorbs to the two strains, although adsorption to Δ*lsr2* cells is slightly better than to wild-type cells (Fig. 3a, and Extended Data Figs. 4 and 5). The reason for this phenotype is unclear, although we note that some BPs host range mutants show substantially enhanced adsorption to *M. smegmatis* mc^2^155 relative to wild-type BPs^47^. N-QTF stains both strains similarly (Fig. 3a).

To test whether Lsr2 influences a post DNA injection process, we utilized reporter phages that express mCherry within the host cell during phage replication^48^ (Fig. 3b). In combination with SYTOX Orange-labelling, this allowed us to sequentially observe adsorption of phage particles and expression from the Fionnbharth-mCherry reporter phage (Fig. 3c). With this imaging strategy, we verify that Fionnbharth adsorption is similar for WT (Supplementary Video 2) and *Δlsr2* cells (Fig. 3c 3rd column from left, Supplementary Video 3 and Extended Data Fig. 5), with all cells in the imaging field bound with multiple phages. After adsorption, the WT cells begin expressing mCherry (Fig. 3c fourth column) and lyse within 24 h. In contrast, only half of the *Δlsr2* cells express mCherry (Fig. 3c, fourth column) and lyse after adsorption (Fig. 3c, fifth column), while the other half continue growing and eventually fill the field (Fig. 3c, fifth column). Fionnbharth thus binds and delivers DNA to WT and Δ*lsr2* cells, consistent with the adsorption observed above (Fig. 3c), but lytic replication is limited in the Δ*lsr2* cells. Phage BPs infects Δ*lsr2* (Supplementary Video 4) and WT (Supplementary Video 5) cells similarly (Fig. 3c bottom row). Lsr2 is thus required for normal lytic growth of Fionnbharth (and probably other Lsr2-dependent phages) but not for BPs and other Lsr2-independent phages (Fig. 1). Δ*lsr2*-mediated resistance occurs via a post-injection mechanism blocking entry into or completion of the lytic life cycle.

### *lsr2* deletion limits epidemic spread of phage

**Extended Data Fig. 6 |:**
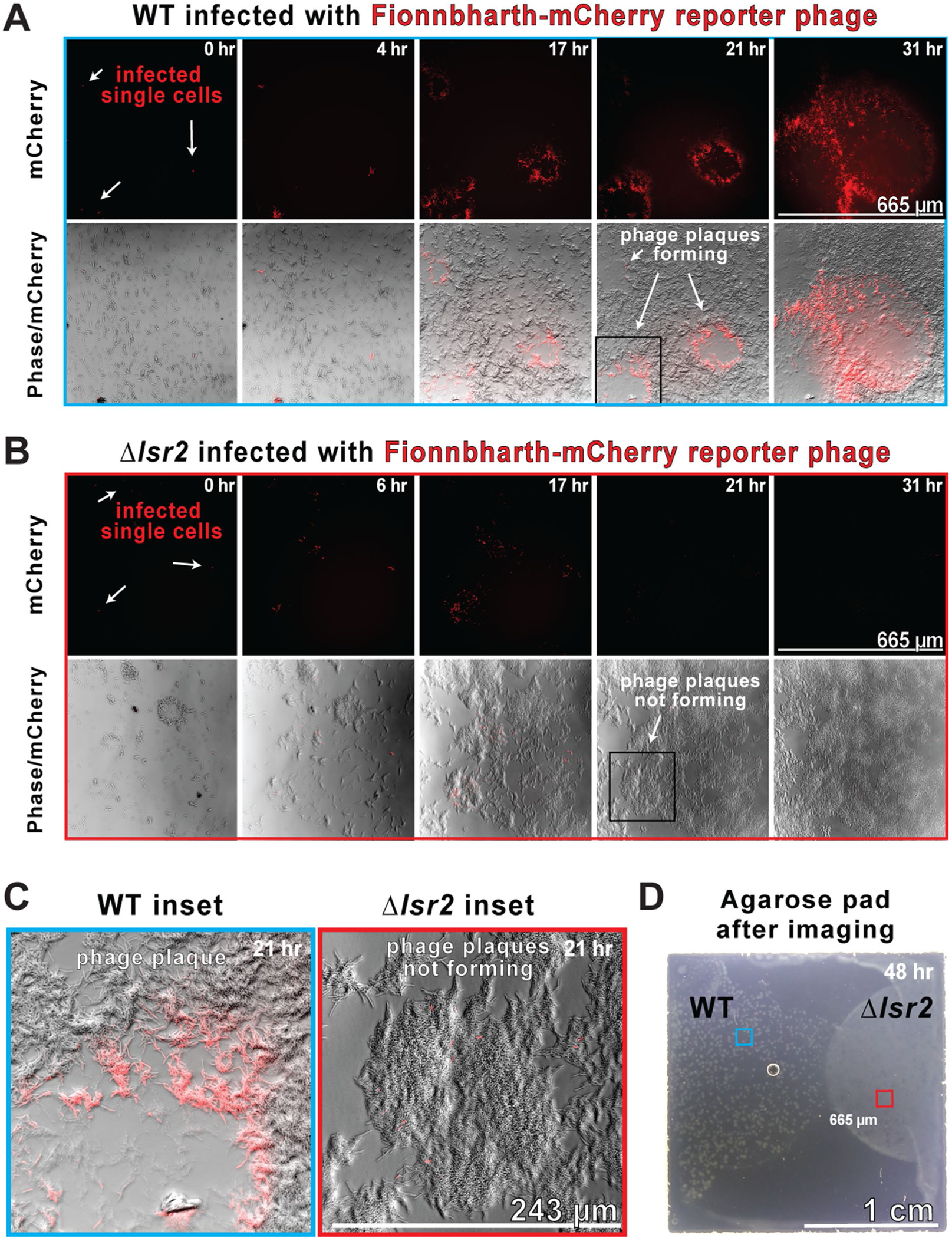
Visualizing phage plaque formation reveals that *lsr*2 deletion limits epidemic spread. (A) WT or (B) Δ*lsr2 M. smegmatis* cells were mixed with “seeder” cells infected with the Fionnbharth-mCherry reporter phage, which become the “single infected cells” noted in the left-most mCherry panels, at a ratio of 1:1000 and 1 μL of WT or Δ*lsr2* cells was spotted onto opposite sides of a 2% agarose 7H9 pad and imaged for 31 hours with a 20x objective to visualize large 665 μm fields. (C) Magnification of insets from panels A and B showing the development of fluorescent phage plaques in WT (panel A) and the lack of plaques in the Δ*lsr2* condition (panel B). Blue and red squares depict the approximate locations of the 665 μm fields shown in panels A & B. (D) A transmitted light photograph of the 2 cm agarose pad used in an epidemic imaging experiment 48 hours after the initiation of the experiment. The WT condition on the left side of the pad displays a web-like phage-plaquing pattern while the Δ*lsr2* condition on the right grows as a homogenous lawn without notable plaques.

To better understand how Δ*lsr2*-mediated resistance operates at the population level, we imaged reporter phage infection of a large field of cells on an agarose pad with a low magnification objective (×20) and time-lapse widefield fluorescence microscopy (Extended Data Fig. 6)^49^. This imaging strategy allowed us to observe plaque formation, zones of clearing approximately 1 mm in diameter, where all bacteria on the pad were lysed by a phage epidemic. WT or Δ*lsr2* cells were infected with Fionnbharth-mCherry reporter phage, washed extensively and diluted 1,000× with uninfected cells before being spotted at high density on agarose pads. In the WT condition, infected single cells express mCherry and lyse, igniting widespread infections that cascade through bacterial colonies creating phage plaques that can be seen with the naked eye (Extended Data Fig. 6 and Supplementary Video 6). While the lawn of WT cells is decimated by the Fionnbharth reporter phage, the Δ*lsr2* cells are effectively protected at a population level, with only initial minor outbreaks that are eventually outcompeted and absorbed into the lawn of growing *M. smegmatis* (Extended Data Fig. 6b–d). There is seemingly an initial burst of infection in a subset of *M. smegmatis* mc^2^155 Δ*lsr2* cells (Extended Data Fig. 5), although subsequent rounds of infections are strongly reduced.

### *lsr2* deletion causes mycobacteriophage assembly defects

**Fig. 4 |:**
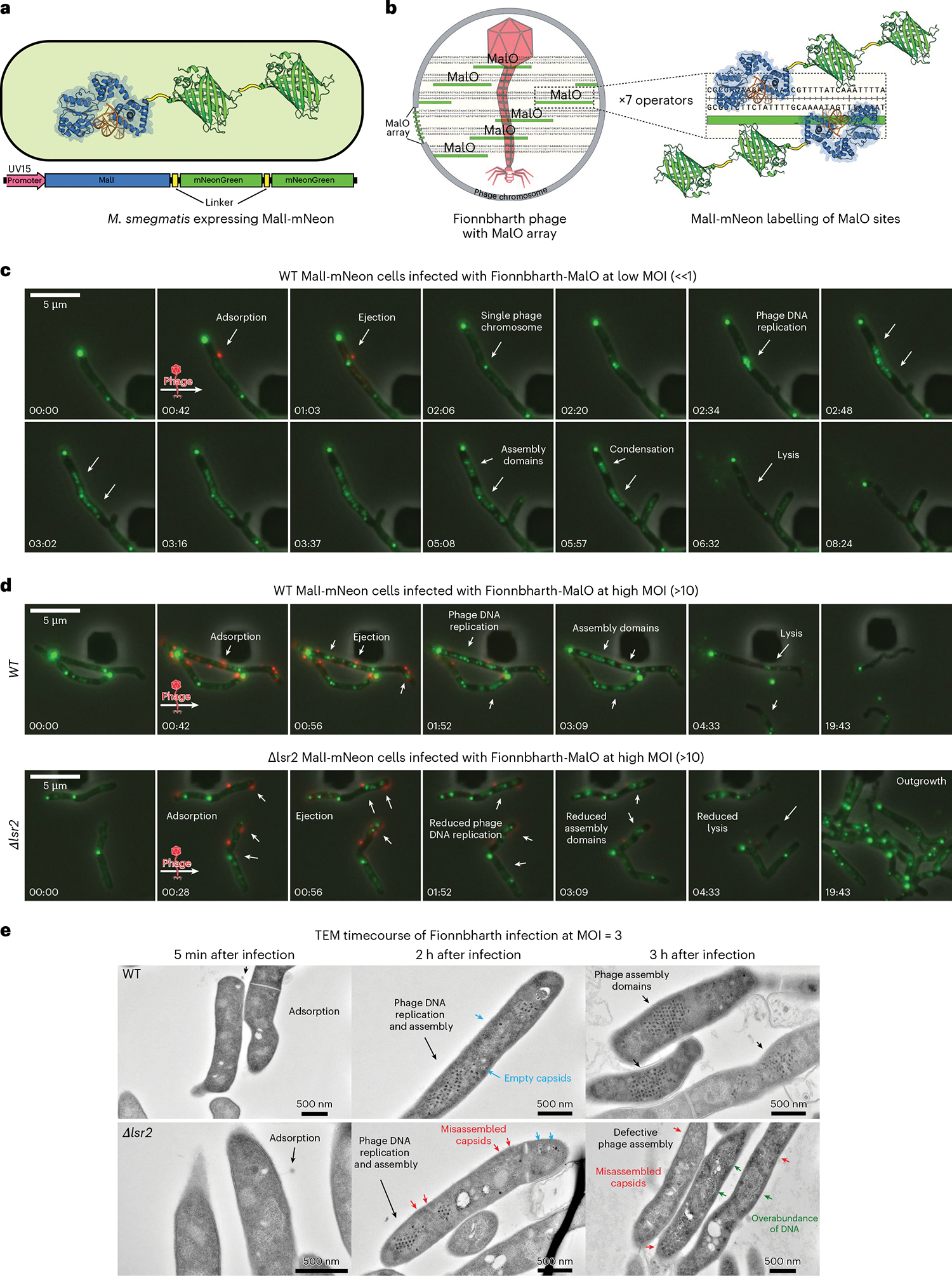
Mycobacteriophage replication and assembly occur in spatially defined domains and *lsr2* deletion causes assembly defects. **A**, Schematic depicting the MalI-mNeon protein construct expressed in *M. smegmatis* cells to visualize the dynamics of phage replication and assembly. The MalI transcription factor was cloned in frame with a high-expressing mycobacterial promoter (UV15) and two copies of the fluorescent mNeonGreen protein joined with short glycine-based linkers. **B**, The Fionnbharth-MalO reporter phage contains 7 MalO (operator) binding sites for the transcription factor MalI. Each operator site can accommodate 2 MalI transcription factors for a total of 14 fluorescent MalI interactions per phage chromosome. **C**, To visualize single phage infection events at the single-cell level, *M. smegmatis* cells grown in a CellASIC microfluidic device were exposed to a short pulse of diluted (10^5^ p.f.u. ml^−1^) SYTOX Orange-stained Fionnbharth-MalO phage. White arrows highlight important events in the infection life cycle. A single phage binding event (red focus, at 00:42) is followed by the formation of a proximal MalI-mNeonGreen focus, consistent with phage ejection and recruitment of MalI-mNeonGreen protein to MalO sites on the infecting phage chromosome. Over the course of infection, the single focus multiplies into many foci that spread out across the interior of the cell and then organize regionally into multiple phage replication domains followed by cell lysis and release of phage particles. **D**, The dynamics of phage replication and assembly were visualized at a high MOI of 10 via a short pulse of concentrated (10^7^ p.f.u. ml^−1^) SYTOX Orange-stained phage particles. Deletion of *lsr2* results in diminished phage replication foci with reduced brightness and a loss of cell lysis. **E**, A Fionnbharth phage infection timecourse of WT (top panels) and Δ*lsr2* (bottom panels) cross-sectioned cells visualized via negative-stain TEM. Early log phase cultures were infected at a MOI of 3 and samples were collected at the indicated timepoints, rapidly fixed, stained, embedded and sectioned for TEM imaging. Arrows indicate significant events in the phage infection life cycle and observed morphological differences between WT and *Δlsr2* infection.

To visualize Fionnbharth genome replication directly, we constructed a lytic recombinant phage carrying an array of seven MalO operator sites that are recognized by the *Escherichia coli* MalI protein (Fionnbharth-MalO phage) (Fig. 4). MalI binds to the MalO array with high affinity and can be observed microscopically using MalI fused via a short glycine linker to mNeonGreen (Fig. 4a)^50^. Each operator site can accommodate two MalI protomers for a total of 14 fluorescent MalI interactions per phage chromosome (Fig. 4b). The MalI-mNeonGreen fusion was cloned downstream of a strong promoter (UV15) on a mycobacterial vector and shows diffuse cytoplasmic fluorescence in uninfected cells, with most cells also containing at least one MalI-mNeonGreen focus near the cell poles (Fig. 4c, panel 1). This polar fluorescence probably represents aggregated protein in inclusion bodies as a consequence of high-level expression^51^. Expression from weaker promoters does not completely eliminate these aggregates and a high level of expression is needed to visualize single phage chromosomes (Fig. 4c). The polar aggregates do not appear to interfere with or alter the dynamics of phage replication or cell growth.

*M. smegmatis* cells harbouring the MalI-mNeonGreen fusion were exposed to a short pulse of diluted (10^5^ plaque-forming units (p.f.u.) ml^−1^) SYTOX Orange-stained Fionnbharth-MalO phage within a CellASIC microfluidic device and monitored via fluorescence microscopy. Single phage binding events were observed (red foci, Fig. 4c panel 2 from left and Supplementary Video 7), followed by formation of a proximal MalI-mNeonGreen focus, consistent with phage ejection and binding of MalI-mNeonGreen protein to MalO DNA sites on an infecting phage genome. As the infection continues, these foci spread across the interior of the cell and organize regionally into multiple phage assembly Domains, followed by cell lysis and release of phage particles. We refer to these as zones of phage DNA replication (ZOPR). The dynamics of phage replication and assembly were also visualized at a higher MOI of 10 in WT (Supplementary Video 8) and Δ*lsr2* cells (Supplementary Video 9) via a short pulse of SYTOX Orange-stained phage particles at 10^7^ p.f.u. ml^−1^ (Fig. 4d). Deletion of *lsr2* results in diminished phage replication foci, brightness and organization, followed by a reduction in the proportion of cells undergoing cell lysis (Fig. 4d). Lsr2 is thus required for the formation of active phage replication domains.

To validate these findings and obtain higher spatial resolution on the lytic phage infection life cycle, WT and *Δlsr2 M. smegmatis* cells were examined by negative-stain TEM. Log phase cultures were infected with Fionnbharth at a MOI of 3 and samples were collected at the indicated timepoints, rapidly fixed, stained, embedded and sectioned for TEM imaging. The timecourse of Fionnbharth infection reveals several morphological differences between WT (Fig. 4e top panels) and *Δlsr2* (Fig. 4e bottom panels) cells. Single phage particles appear as dark electron-dense spheroids^52^, and late in infection of WT *M. smegmatis*, hundreds of phage capsids are ordered into a highly compacted pseudo crystal lattice (Fig. 4e). The electron micrographs mirror what was observed with fluorescence microscopy; Fionnbharth begins forming tightly organized phage assembly domains 2–3 h after infection and *lsr2* deletion results in reduced mature phage capsid formation. *Δlsr2* infected cells contain fewer electron-dense capsids and more empty and misassembled capsids (Fig. 4e).

Deletion of *lsr2* has numerous morphological consequences that have been observed at the colony and biofilm level as well as the cellular level^53^. Cells lacking Lsr2 are slightly shorter and wider and have altered DNA replication dynamics^53^. Our observations of the inner workings of *Δlsr2* cells via TEM are consistent with these findings. We saw more evidence of bulky DNA in *Δlsr2* cells than in WT cells, suggesting an overabundance of uncompacted host chromosomal DNA. This deficit in DNA organization could lead to gross morphological defects as well as less efficient or hampered assembly of phage capsids.

### Lsr2 re-localizes to zones of phage DNA replication

**Fig. 5 |:**
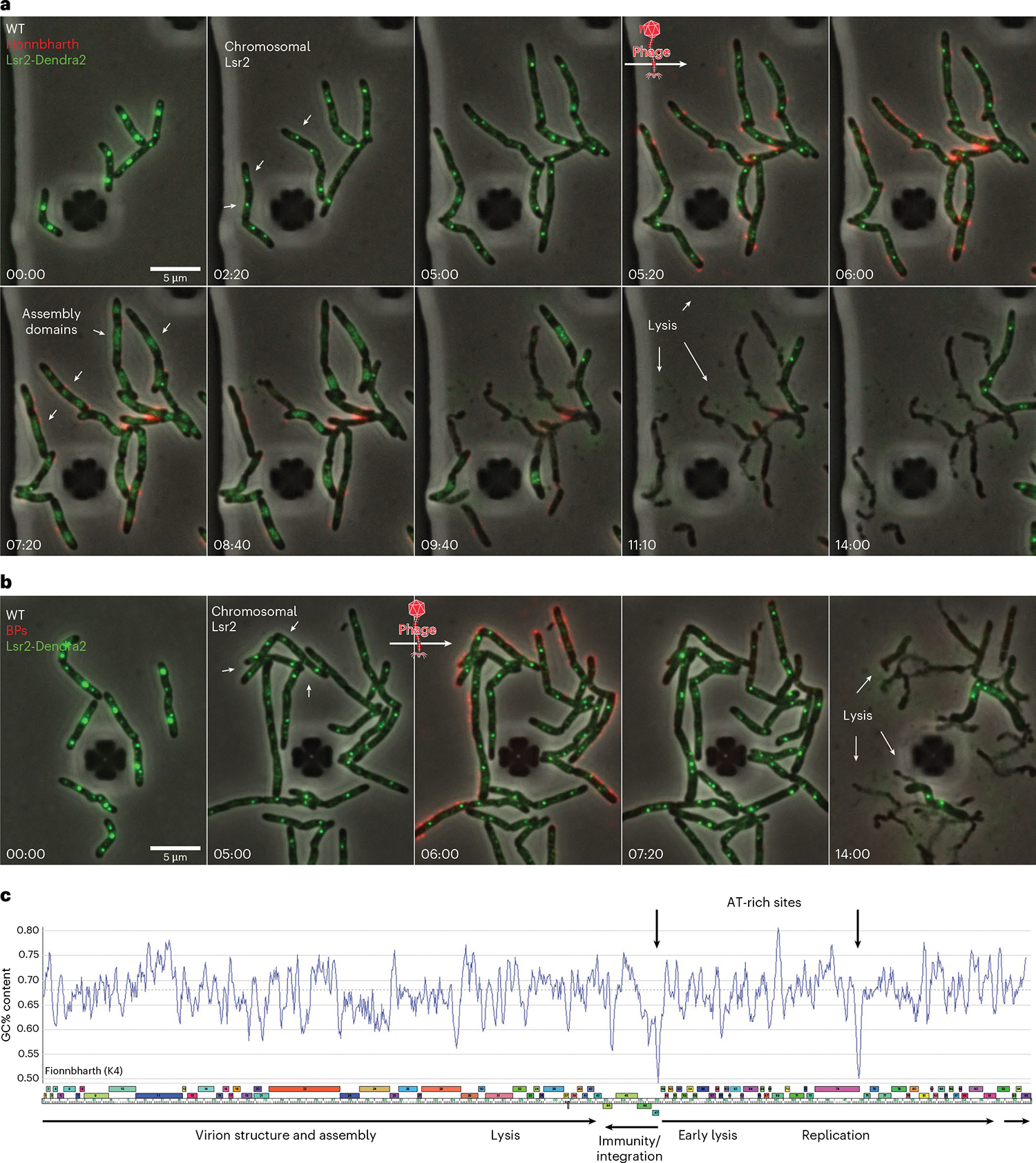
Re-localization of Lsr2 to zones of phage DNA replication. **a**,**b**, Time-lapse of Lsr2-Dendra2 *M. smegmatis* cells grown and infected with fluorescent Fionnbharth (**a**) or BPs (**b**) phages in a CellASIC microfluidic device. Cells were infected via a 1 h pulse of SYTOX Orange-labelled phage particles at 10^7^ p.f.u. ml^−1^. Lsr2-Dendra2 forms nucleoprotein complexes on the host chromosome, these complexes are seen as discrete, dynamic foci near the DNA replication machinery. Upon phage infection with Fionnbharth, Lsr2 foci rapidly disintegrate and re-localize into the Fionnbharth ZOPR (**a**). This dynamic redistribution of Lsr2 protein does not occur with infection of Lsr2-insensitive phages like BPs (**b**). White arrows highlight important events in the infection life cycle. **C**, A genome map of Fionnbharth with GC% content displayed on the *y* axis. Bold arrows indicate two regions with notably lower GC% content: one in the intergenic region between the divergently transcribed repressor and *cro*-like genes, and a second immediately downstream of a putative DNA primase.

To better understand the spatiotemporal dynamics of Lsr2 during phage infection, we imaged an *M. smegmatis* strain in which endogenous Lsr2 is tagged with the fluorescent protein Dendra2. This strain was recently used to visualize the chromosomal localization of Lsr2 during the mycobacterial life cycle^53^. Lsr2 forms nucleoprotein complexes on the host chromosome, these complexes are visible as discrete, dynamic foci near the DNA replication machinery. Upon infection with Fionnbharth, Lsr2 foci rapidly disintegrate and re-localize into the phage ZOPR (Fig. 5a and Supplementary Video 10). This dynamic redistribution of Lsr2 protein does not occur with infection of Lsr2-insensitive phages like BPs (Fig. 5b and Supplementary Video 11). These data suggest that Lsr2 either directly or indirectly associates with the Fionnbharth chromosome and that these interactions may be important for replication and assembly of phage DNA. Overall, the Fionnbharth genome has a GC% content of 67.4%, similar to its *M. smegmatis* host. However, there are two regions notably lower in GC% content (Fig. 5c): one in the intergenic region between the divergently transcribed repressor and *cro*-like genes (genes *47* and *48*), and a second immediately downstream of a putative DNA primase (gene *74*) (Fig. 5c). These are plausible regions of Lsr2 binding. We note, however, that most mycobacteriophage genomes, including BPs, show variations in GC% content and regions with lower GC% content.

### Phage-encoded *lsr2* does not impact lytic infection

**Fig. 6 |:**
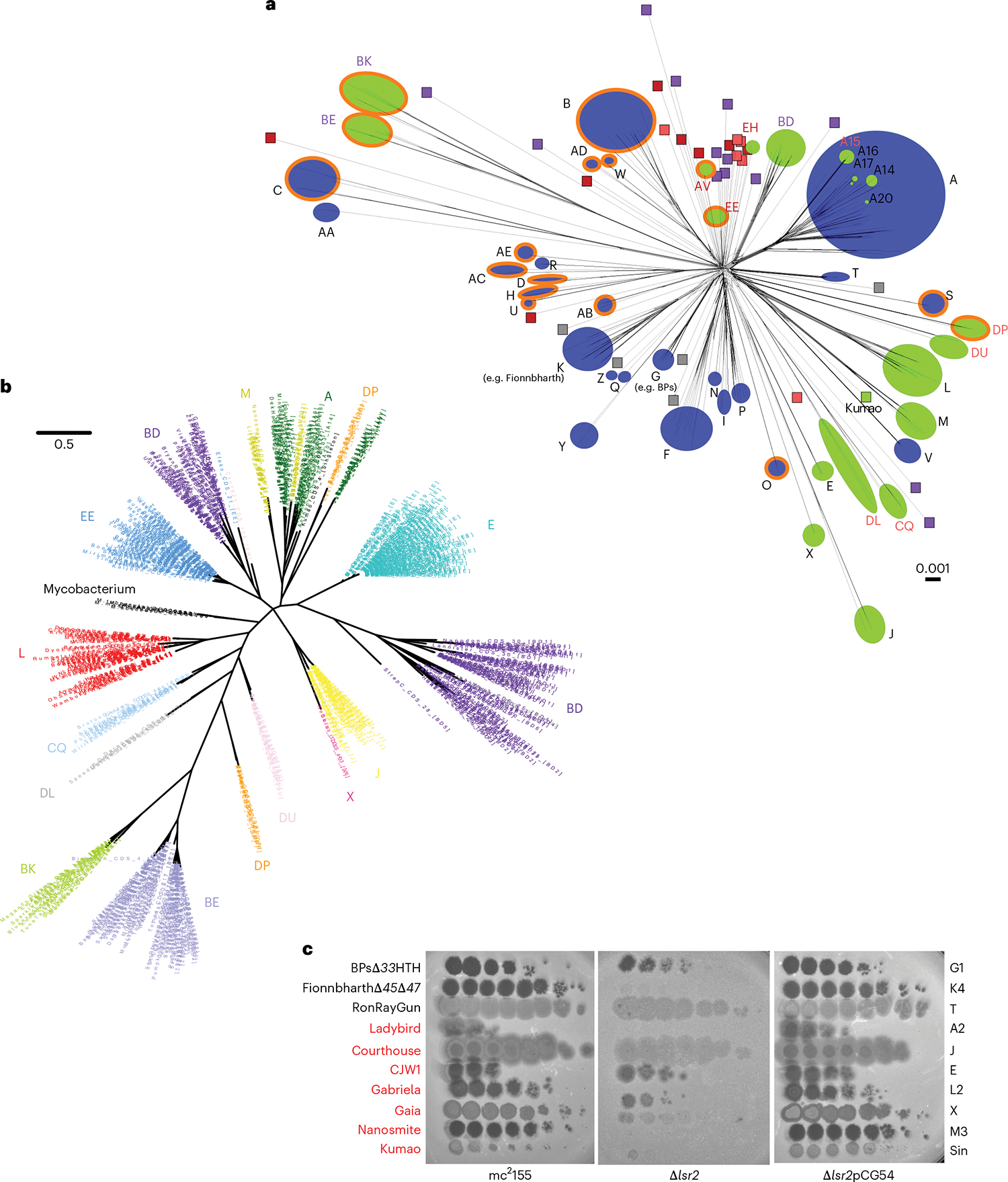
Actinobacteriophage Lsr2 diversity. **A**, A network phylogeny of actinobacteriophages, including phages of *Gordonia*, *Microbacterium*, *Mycobacterium* and *Streptomyces*, based on shared gene content^73^ and constructed using Splitstree^67,73^. The tree was constructed using up to three members of each subcluster or non-divided cluster, and clusters are indicated as circles with their designations (A, B and so on). Green and blue circles represent phage clusters that carry or lack lsr2 homologs, respectively. Cluster text designations are coloured according to host species: *Gordonia* (pink), *Streptomyces* (purple), *Microbacterium* (red) and *Mycobacterium* (black/dark grey). Singletons are represented by small boxes, similarly coloured by cluster designation. Clusters of lytic phages have an orange outer ring, all others are temperate. Hosts and life cycles of singleton phages are not shown for simplicity but are available at https://phagesdb.org. Kumao (*Mycobacterium*; temperate) is the only singleton carrying *lsr2*. Scale bar indicates pairwise hamming distance^74^. **b**, A maximum likelihood phylogenetic tree of *lsr2* in actinobacteriophages. All actinobacteriophages with an *lsr2* homologue are shown as well as the *lsr2* genes of *M. smegmatis*, *M. tuberculosis*, *M. abscessus, Mycobacterium kansassii* and *Mycobacterium leprae*. Cluster designations are shown. Scale bar indicates nucleotide substitution/site. **c**, Role of host Lsr2 in infection of *lsr2*-containing phages. Phage names are shown in black or red type indicating the absence or presence of a canonical (113–153 residues) *lsr2* gene in the phage genome. Phage names marked in red possess *lsr2*, while phage names in black are phages that completely lack an *lsr2* homologue. Phage lysates were tenfold serially diluted and spotted onto lawns of *M. smegmatis* mc^2^155, *M. smegmatis* Δ*lsr2* and the complementation strain *M. smegmatis* Δ*lsr2*pCG54. Phage names are shown at the left, and their cluster/subcluster/singleton (sin) designations are shown at the right.

Finally, we note that *lsr2* homologues are present in many actinobacteriophage genomes including those of *Mycobacterium*, *Gordonia*, *Streptomyces* and *Microbacterium*^54,55^ (Fig. 6a), raising the possibility that phages encode these to counter host defence mechanisms involving loss of Lsr2 activity. These phage-encoded *lsr2*-like genes span substantial sequence diversity and many are distantly related to host *lsr2* genes (Fig. 6b). Moreover, they are present in the genomes of both lytic and temperate phages (Fig. 6a), and for at least some temperate phages including Ladybird (Cluster A2), they are known to be lysogenically expressed^56^. In those instances, including all of the *lsr2*-containing *Mycobacterium* phages, they are unlikely to confer a counter-defence mechanism. In some lytic phages (for example, Clusters AV and BE infecting *Microbacterium* and *Streptomyces*, respectively), Lsr2 is encoded within long terminal repeats, and in many lytic phages (for example, Clusters BD and BK infecting *Streptomyces*, and EE and EH infecting *Microbacterium*), the protein is shorter than 80 residues, primarily spanning the N-terminal oligomerization domain and lacking the DNA binding domain. These ‘truncated’ Lsr2 proteins are more likely to act via dominant negative interactions than by complementation of host *lsr2* loss. We tested several *lsr2*-carrying mycobacteriophages (all have full-length *lsr2* genes) for their response to deletion of the host *lsr2* (Fig. 6c). In several, the efficiency of plaquing is greatly reduced, indicating that these phage-encoded *lsr2* genes do not compensate for host *lsr2* loss. An alternative explanation is that phage *lsr2* genes act in inter-phage competition and interfere, perhaps through dominant negative interactions, with phage superinfection of lysogens, or by exclusion in lytic growth. We note that neither Fionnbharth nor any other Cluster K phages carry their own *lsr2*.

## Discussion

Here we show that Lsr2 plays an important role in productive infection of many mycobacteriophages. Loss of Lsr2 influences a broad variety of mycobacteriophages, manifested as resistance—that is, a sharp reduction in the efficiency of plating—or revealed as more subtle impacts such as reduced plaque size or increased turbidity. Lsr2 is known to play a role in organizing and maintaining host DNA replication systems^53^, and similarly plays a role in organizing phage zones of replication.

A particularly notable observation is the finding that for the set of genomically diverse phages we examined, all preferentially bind at sites of new cell synthesis and not at regions of ‘old’ cell wall. This has several important implications. First, it suggests that phages may have evolved to specifically recognize cells that are actively growing and are thus metabolically active, to support phage replication. Second, it provides spatial information to coordinate phage infection with other structurally organized cellular components that might be needed for phage replication. Third, it suggests a specific mechanism that phages can exploit to outcompete other phages in a competitive environment.

For example, a newly infecting phage undergoing lytic growth could prevent superinfection (and potential theft of valuable resources needed for reproduction) simply by inhibiting cell wall biosynthesis, for which there are many plausible targets. We note that mycobacteriophage Fruitloop interferes with superinfection by phage Rosebush by expressing a protein (gp52) that binds to and inactivates DivIVA which is required for cell wall biosynthesis^57^. Many mycobacteriophage proteins expressed during the lytic cycle are toxic for mycobacterial growth and often cause division or morphological defects, consistent with disruptions in cell wall synthesis^58^.

Exquisite TEM data collected in 1961 recorded “groups of clustered mycobacteriophage particles” within sectioned cells of the *Mycobacterium* Jucho strain^52^. Our observations are remarkably consistent with these studies and contribute a temporal dimension that brings these dynamic structures to life. Mycobacteriophage ZOPR are evidently not compartmentalized similar to the pseudo-nuclear structures described for *Pseudomonas* large phages^59^ and may be defined by the available space for DNA replication in an otherwise crowded cell. Nonetheless, the ZOPR are distinct from the sites of host DNA replication, as reflected in the re-organization of Lsr2 during phage infection. Cryo-electron tomography and super-resolution microscopy will be useful approaches for further defining the mycobacteriophage ZOPR. We show that host Lsr2 protein is required for establishing these zones and without it, phage replication is impaired, leading to defective epidemic spread (Extended Data Fig. 6 and Supplementary Video 6). Curiously, some phages (for example, Bxb1, RonRayGun) have only minor reductions in efficiency of plaquing in the absence of Lsr2 and presumably employ different strategies for replication and lytic growth.

The newly developed tools described here, both for visualizing phage infection and constructing informative phage derivatives, are important for understanding phage–host dynamics in mycobacteria and should be broadly applicable. The combination of SYTOX Orange-stained phage particles and the N-QTF probe for newly synthesized mycolic acids shows the remarkable preference of phages to adsorb to regions of new cell wall synthesis. This may be a general phage strategy and these tools will be valuable for further exploring this question. The mycobacteriophage engineering methods^48,60^ will also be generally applicable, especially combining mCherry reporter phages showing phage gene expression and MalO recombinant phages that reveal phage DNA localization.

Elucidating mycobacteriophage resistance mechanisms is relevant to their potential therapeutic use. In *M. tuberculosis*, for which FionnbharthΔ*45*Δ*47* is a therapeutic candidate^18^, both domains of Lsr2 are essential for bacterial viability^28^, minimizing the prospects for *lsr2* loss as a potential resistance mechanism. Interestingly, in *M. abscessus*, as in *M. smegmatis*, *lsr2* is non-essential^34,61^, although its loss considerably diminishes *M. abscessus* virulence. *M. abscessus lsr2* has not been identified as a phage resistance target in vitro^22^ or in vivo^62^, including with the therapeutically useful phage Muddy, and this may reflect a beneficial trade-off between phage sensitivity and pathogenicity.

## Methods

### Bacterial strains and media

Liquid cultures of *M. smegmatis* mc^2^155 were grown in Middlebrook 7H9 media and were used to propagate the phages used in this study. An unmarked deletion of *lsr2* (*M. smegmatis* GWB142) was constructed using the recombineering plasmid pJV53 and electroporation of an allelic exchange substrate with flanking homology to *lsr2*^63^.

### Isolation of phage-resistant mutants

Six independent 1 ml cultures of 1 × 10^8^ colony-forming units (c.f.u.) *M. smegmatis* mc^2^155 were incubated with 10 μl of lysates containing 10^8^–10^9^ p.f.u. phage and incubated with shaking at 250 r.p.m. at 37 °C for 32 h. Subsequently, 75–150 μl aliquots were then spread on Middlebrook 7H10 solid media and incubated at 37 °C for 3 d or until isolated phage-resistant colonies were visible. Phage-resistant candidate strains were purified by streaking twice on Middlebrook 7H10 and used to inoculate 3 ml cultures of Middlebrook 7H9 with ADC (50 g l^−1^ albumin fraction V Cohn Analog (Lampire Biologicals), 20 g l^−1^ dextrose (Fisher), 8.5 g l^−1^ NaCl) and 0.05% Tween 80. After growth to saturation, cultures were used to prepare bacterial lawns and serial dilutions of phages were spotted onto the lawns to determine phage susceptibilities.

### DNA extraction and sequencing of bacterial strains

DNA was isolated from the parent strain and phage-resistant mutants for DNA sequencing. Briefly, 1 ml of cell culture was lysed, pelleted and then resuspended in Nuclei lysis solution (Promega). The cell resuspension was added to a tube containing lysing matrix B (MP Biologicals) and milled three times with intermittent incubation on ice. Phenol-chloroform-isoamyl alcohol was then added and the aqueous phase was removed. DNA was precipitated using isopropanol and 3 M sodium acetate.

The parent strain was then sequenced by both Illumina MiSeq and Oxford Nanopore MinIon technologies, and a hybrid assembly using long and short reads was subsequently performed with Unicycler^64^. The genome was checked for completeness and accuracy, then corrected using Consed^65,66^. Mutant strains were sequenced by Illumina MiSeq only and resulting reads were aligned to the completed parent strain, again using Consed. An in-house programme called AceUtil was used to identify differences between the mutant reads and the parent genome, and all mutations were confirmed by close inspection of the reads.

### Construction of complementation plasmids pCG52, pCG54 and pCG67

Vectors pCG52 and pCG54 were constructed by PCR linearization of vectors pLO73 and pLO76, respectively^43^, removing the mCherry gene in the process. Insertion of *lsr2* into the vectors was done using a gblock (Integrated DNA Technologies) containing *lsr2* flanked by 20 bp upstream and downstream homology to pLO73 and pLO76 using NEBuilder (New England Biolabs), following the manufacturer’s recommendations (Supplementary Table 1). The plasmid pCG67 was similarly made by inserting an *lsr2* gblock with 20 bp upstream and downstream homology to pLO76, substituting guanine at position 303 for adenosine, resulting in a G100A amino acid substitution at the Lsr2 AT-hook core site in the DNA binding domain (Supplementary Table 1).

### Phylogenetic analyses

Splitstree^67^ was used to represent a network phylogeny of Actinobacteriophages. Up to three phage representatives per cluster, subcluster or singletons were used where available for phages infecting *Gordonia, Streptomyces, Microbacterium* and *Mycobacterium*. A maximum-likelihood phylogenetic tree was constructed using Qiagen CLC genomics workbench v22.0 with the GTR model, four substitution rate categories and 100 replicates. Alignments for maximum-likelihood phylogeny were made using Aliview^68^, with the option to translate nucleotide sequences to amino acid. Amino acid alignments of Mycobacterial Lsr2 were done using Qiagen CLC genomics workbench v22.0.

### Adsorption and one-step growth curve experiments

To assess adsorption,1 ml cells of either WT mc^2^155 or Δ*lsr2* freshly grown to saturation were used to inoculate 10 ml 7H9 ADC and grown for 4 h at 37 °C, with shaking at 250 r.p.m. until reaching an optical density (OD) of ~0.2 or approximately 7 × 10^7^ c.f.u. ml^−1^. After taking OD measurements, cells were pelleted and resuspended in 1 ml 7H9 with 1 mM CaCl_2_ to a 10-fold increase in cell concentration. Phage were added to cells at a MOI of 0.0001, approximately 10^5^ p.f.u. ml^−1^. Cells were incubated at 37 °C with shaking at 250 r.p.m. Every 10 min for 1 h, aliquots were taken, cells were pelleted, and supernatants were diluted 10-fold and plated on mc^2^155 to assess plaque forming units. Experiments were done in biological triplicates.

For one-step growth curves, 50 μl of either WT mc^2^155 or Δ*lsr2* freshly grown to saturation were used to inoculate 10 ml 7H9 ADC and grown for ~16 h at 37 °C. Cells at log phase were diluted to OD of ~0.2. After taking OD measurements, cells were pelleted and resuspended in 1 ml 7H9 with 1 mM CaCl_2_ to a 10-fold increase in cell concentration. Phage were added to cells at a MOI of 0.001, approximately 10^6^ p.f.u. ml^−1^. Cells were incubated at 37 °C with shaking at 250 r.p.m. Aliquots were removed at specific times, serially diluted 10-fold between 10^−1^ and 10^−4^ and plated on mc^2^155 cells to assess plaque forming units at each timepoint. Experiments were done in biological triplicates.

### Construction of MalO reporter Fionnbharth

A DNA cassette containing seven Mal Operator sites was amplified from plasmid pCB182 using primers MalO cassette amplify F (5’-TCTGCTCGAGGAATTCTCCAGATTCTAGTG 3’) and MalO cassette amplify R (5’-GTAGCCATGCAGATGACCTACTCCCTGATT 3’) with Q5 polymerase 2X Master Mix (New England Biolabs). Two gBlocks (Supplementary Table 1) containing homology to 280 bp upstream of Fionnbharth gene *45* and 429 bp downstream of Fionnbharth gene *47* were used to build a larger substrate in which they flank a MalO cassette, constructed by PCR using primers with 18 bp of homology to the two gblocks. A linear substrate was assembled using NEBuilder to join the gBlocks and the MalO cassette, and the entire substrate was amplified using primers mal-Fionn-F (5’-AACATAGTCCAGATTTATGGACAAAGCAACTCG 3’) and mal-Fionn-R (5’-CGGCCGGTACTCCTACCAAGCACTACACAG 3’) with Q5 2x Master Mix (New England Biolabs). The recombinant phage was then made using CRISPY-BRIP^48^. Briefly, the amplified substrate was purified by gel extraction and electroporated into *M. smegmatis* mc^2^155pJV138, recombineering competent cells together with Fionnbharth genomic DNA. The mixture was then plated with a culture of *M. smegmatis* mc^2^155 containing plasmid pCCK510 with a single guide RNA targeting Fionnbharth gene *45*^48^, and plated on 7H10 solid medium containing 100 nM Anhydrotetracycline to select for mutants containing the allelic replacement. Candidate recombinant phages were screened using two rounds of PCR.

### Microscopy

For all imaging experiments, *M. smegmatis* mc^2^155 was sub-cultured in liquid Middlebrook 7H9 media supplemented with 5 g l^−1^ albumin, 2 g l^−1^ dextrose, 0.85 g l^−1^ NaCl, 0.003 g l^−1^ catalase, 0.2% (v/v) glycerol and 0.05% (v/v) Tween 80. Before imaging, *M. smegmatis* mc^2^155 was sub-cultured three times with the above complete 7H9 with 1 mM CaCl_2_ media lacking Tween 80 to give the bacteria time to build their capsule, which is required for phage attachment. To prevent clumping, cultures grown without Tween 80 were subjected to high-speed shaking at 200–250 r.p.m. in inkwell culture vessels.

Phase-contrast and epifluorescence images were collected with a widefield Nikon Eclipse Ti-E inverted microscope equipped with an Okolab Cage incubator warmed to 37 °C with Cargille Type 37 immersion oil. A Nikon CFI Plan Apo DM Lambda ×100 1.45 NA oil objective and a Nikon CFI Plan Apo DM Lambda ×20 0.75 NA objective were used with Perfect Focus System for maintenance of focus over time. N-QTF, Dendra2, mCherry2B and SYTOX Orange nucleic acid stain (ThermoFisher) were excited with a Lumencor Spectra X light engine with Chroma FITC (470/24) (for N-QTF and Dendra2) and mCherry (575/25) (for mCherry2B and SYTOX Orange) filter sets, respectively, and collected with a Spectra Sedat Quad filter cube ET435/26M-25 ET515/30M-25 ET595/40M-25 ET705/72M-25 (for N-QTF and Dendra2) and a Spectra CFP/YFP/mCherry filter cube ET475/20M-25 ET540/21M-25 ET632/60M-25 (for mCherry2B and SYTOX Orange). Images were acquired with an Andor Zyla 4.2 sCMOS controlled by NIS Elements software. For time-lapse experiments, images were collected every 10–12 min (unless specified otherwise) via ND acquisition using an exposure time of 100 ms or 200 ms and 50% or 100% illumination power for fluorescence. Multiple stage positions (fields) were collected using the default engine TiZ.

### Image analysis

Fields best representing the overall experimental trend with the least technical artefacts were chosen for publication. Gamma, brightness and contrast were adjusted (identically for compared image sets) using FIJI^69^.The FIJI plug-ins Stack Contrast^70^ and StackReg^71^ were used for brightness matching and registering image stacks. Phase-contrast images were segmented using DeepCell^46^ and analysed using a custom MATLAB programme. Briefly, peaks were located in image profiles in the red (SYTOX) and green (N-QTF) channels along lines perpendicular to the segmented cells, and the image background was measured where no cells were present. Peaks in the red channel that were more than one standard deviation above the measured background fluorescence intensity were called ‘phage proximal’. The N-QTF signal was background subtracted and the maximum intensity along lines that were phage proximal was measured.

### Generation of fluorescent phages with nucleic acid stain

Concentrated phage stocks (200 μl, 10^10^–10^11^ p.f.u. ml^−1^) were stained with SYTOX Orange nucleic acid stain^44^. Stained phages were washed four times in 15 ml of phage buffer (10 mM Tris, pH 7.5, 10 mM MgSO_4_, 68.45 mM NaCl, 1 mM CaCl2) using Amicon Ultra-15 centrifugal filter units. After staining, the titre and viability of phages were immediately assessed by plaque assay, and once stained, phages were used for no longer than 1 week as the viability decreased over time. For use in microfluidic experiments, SYTOX Orange-stained phages were normalized to a titre of approximately 10^7^ p.f.u. ml^−1^.

### Fluorescence microscopy with agarose pads

Middlebrook 7H9 media with 2% agarose pads were prepared by mixing one part 10× 7H9 concentrate (which contained 7H9 powder and glycerol at 10× concentrations), one part albumin, dextrose and catalase (ADC) and eight parts of low-melt 2.4% agarose, and mounting on MatTek dishes (No. 1.5 coverslip, 50 mm, 30 mm glass diameter, uncoated). *M. smegmatis* mc^2^155 strains were grown to OD_600_ of ~1.0 (corresponding to 3.5 × 10^8^ c.f.u. ml^−1^) in 7H9 + 1 mM CaCl_2_ without Tween 80 at 37 °C with shaking (200 r.p.m.) and, where required, diluted in media to achieve the desired cell density on the agarose pad. To create the infected seeder cells, 100 μl of normalized bacterial cultures at OD_600_ = 0.5 were infected with the Fionnbharth-mCherry reporter phage at room temperature for 10 min with phage stocks at 10^9^ p.f.u. ml^−1^ to a MOI of 100. Subsequently, infected cells were washed 3× with ice-cold phage buffer (10 mM Tris, pH 7.5, 10 mM MgSO_4_, 68.45 mM NaCl, 1 mM CaCl_2_) to reduce the concentration of un-adsorbed free phage, followed by one wash with ice-cold 7H9 media + 1 mM CaCl_2_ and without Tween 80. Seeder cells were diluted 1:1,000 with uninfected cells before being spotted on agarose pads. This ratio of uninfected to infected cells was optimized such that in randomly chosen microscopy fields (without previous knowledge of which cells in the field were infected), there was likely to be at least one infected cell (Fig. 4). Chilled cells (1 μl) were spotted onto opposite sides of an agarose pad (two strains were imaged on the same pad) and inverted onto the MatTek imaging dish. To prevent formation of small condensation droplets on the lid of the dish, the underside of the lid was soaked with a solution of 0.05% Triton-X-100 in 20% ethanol for 1 min and then allowed to dry. Phase-contrast and fluorescence images were collected every 12 min for 36 h using the ×20 objective.

### Fluorescence microscopy using microfluidic infection

The CellASIC ONIX2 system from EMD Millipore with B04A plates was used for microfluidic imaging experiments (Figs. 2, 3 and 5). Phages used in microfluidic infection experiments were stained with SYTOX Orange nucleic acid stain as described above^44^. *M. smegmatis* mc^2^155 strains were grown to OD_600_ of ~1 (corresponding to 3.5 × 10^8^ c.f.u. ml^−1^) in 7H9 media with 1 mM CaCl_2_ and without Tween 80 at 37 °C with shaking (250 r.p.m.) before being diluted tenfold and loaded into CellASIC B04A plates using the pressure-driven method according to the manufacturer protocol for bacterial cells. The slanted chamber of the plate immobilizes the cells but allows media to flow continuously. First, cells were equilibrated with constant 7H9 media with 1 mM CaCl_2_ and without Tween 80 at a flowrate of 2 psi for approximately 1 h. Second, cells were stained with a constant flow of the N-QTF probe at a concentration of 500 nM in 7H9 media with 1 mM CaCl_2_ and without Tween 80 for 1 h. Next, phages suspended at 10^7^ p.f.u. ml^−1^ in 7H9 media with 1 mM CaCl_2_, without Tween 80 and with 500 nM N-QTF probe were pulsed over the cells for 1 h. For phage infection experiments requiring very low MOI (≪1), SYTOX Orange-stained phage stocks were diluted to 10^5^ p.f.u. ml^−1^. For phage infection experiments requiring high MOI, SYTOX Orange-stained phage stocks were employed at 10^7^ p.f.u. ml^−1^. Finally, cells were grown under constant flow of 7H9 media with 1 mM CaCl_2_, without Tween 80 and with 500 nM N-QTF probe for the duration of the experiment. Microfluidic experiments typically lasted 24 h, after which time uninfected or phage-resistant cells outgrew the chamber. Phase-contrast and fluorescence images were collected every 10–12 min. *M. smegmatis* mc^2^155 cells were stained with fluorescent d-amino acids (FDAA) (Fig. 2c)^72^.

### Negative-stain TEM

*M. smegmatis* mc^2^155 strains were sub-cultured three times in 7H9 with 1 mM CaCl_2_ and without Tween 80 at 37 °C with shaking (200 r.p.m.). Next, 100 ml cultures were grown to OD_600_ of ~1 before being filtered through a 10 μm syringe filter (Acrodisc syringe filter, 10 μm with Versapor membrane). Filtered cells were centrifuged and pelleted at 5,000 *g* for 10 min before phage infection, and cell densities were normalized via resuspension in 1 ml 7H9 media with 1 mM CaCl_2_ and without Tween 80. Cells were infected with phage stocks at 10^11^ p.f.u. to a final MOI of 3, 10 or 100 depending on the experiment. Infected cells were then incubated at 37 °C with shaking (200 r.p.m.) for 5 min before the Eppendorf tubes holding cells were plunged into ice to pause phage development in preparation for imaging at the Harvard Medical School EM Facility. Five microlitres of samples were adsorbed for 1 min to carbon-coated grids (EMS, CF400-CU) that had been made hydrophilic by a 20 s exposure to a glow discharge (25 mA). Excess liquid was removed with a filter paper (Whatman No. 1), the grid was then floated briefly on a drop of water (to wash away phosphate or salt), blotted again on a filter paper and then stained with 0.75% uranyl formate (EMS, 22451) or 1% uranyl acetate (EMS, 22400) for 20–30 s. After removing the excess stain with a filter paper, the grids were examined in a JEOL 1200EX transmission electron microscope or a TecnaiG^2^ Spirit BioTWIN and images were recorded with an AMT 2k CCD camera. TEM imaging experiments were performed twice and micrographs best representing the overall experimental trend with the least technical artefacts were chosen for publication.

To visualize the inner workings of phage-infected cells, thin sections of embedded cells were examined via TEM. *M. smegmatis* mc^2^155 and Δlsr2 strains were sub-cultured three times in 7H9 with 1 mM CaCl_2_ and without Tween 80 at 37 °C with shaking (200 r.p.m.), and were then normalized to OD_600_ = 0.7 (2 × 10^8^ c.f.u. ml^−1^) before being infected at MOI = 3. Samples were collected at indicated timepoints and fixed with 2.5% glutaraldehyde, 1.25% paraformaldehyde and 0.03% picric acid in 0.1 M sodium cacodylate buffer (pH 7.4), followed by centrifugation at 5,000 *g* for 10 min. A pellet of cells was fixed for at least 2 h at room temperature in the above fixative, washed in 0.1 M cacodylate buffer and post-fixed with 1% osmium tetroxide (OsO4)/1.5% potassium ferrocyanide (KFeCN6) for 1 h. This was followed by washing twice in water, once in maleate buffer (MB), incubation in 1% uranyl acetate in MB for 1 h, 2 washes in water and subsequent dehydration in grades of alcohol (10 min each: 50%, 70%, 90%, and 2 ×10 min 100%). The samples were then put in propyleneoxide for 1 h and infiltrated overnight in a 1:1 mixture of propyleneoxide and TAAB Epon embedding resin (TAAB, https://taab.co.uk). The following day, the samples were embedded in TAAB Epon and polymerized at 60 °C for 48 h. Ultrathin sections (about 60 nm) were cut on a Reichert Ultracut-S microtome, picked up on copper grids stained with lead citrate and examined in a JEOL 1200EX transmission electron microscope or a TecnaiG^2^ Spirit BioTWIN; images were recorded with an AMT 2k CCD camera.

### Flow cytometry

*M. smegmatis* mc^2^155 strains were sub-cultured three times in 7H9 with 1 mM CaCl_2_ and without Tween 80 at 37 °C with shaking (200 r.p.m.). Next, 5 ml cultures were grown to OD_600_ of ~1 (~3.5 × 10^8^ c.f.u. ml^−1^) before being filtered through a 10 μm syringe filter. Filtered cells were centrifuged and pelleted at 5,000 *g* for 10 min before phage infection, and cell densities were normalized to OD_600_ = 0.1 via resuspension with 7H9 media with 1 mM CaCl_2_ and without Tween 80. Cells were infected with SYTOX Orange-stained mycobacteriophages at 10^9^ p.f.u. ml^−1^ to MOI = 100 and incubated at room temperature in the dark for 5 min. The Eppendorf tubes holding cells were then plunged into ice to pause phage development in preparation for flow cytometry. Cells were stained with the N-QTF probe at a concentration of 500 nM, kept on ice and protected from light before flow cytometry analysis. Cells were analysed by flow cytometry on a MACSQuant (VYB excitation: 488 nm, 561 nm; emission filter: 525/50, 615/20). Bacterial cells were gated on SSC-A/FSC-A and an unstained sample was used as a negative control to draw positive gates for N-QTF and SYTOX Orange fluorochromes (Extended Data Fig. 5). More than 50,000 events were recorded. Data were analysed using FlowJo.

## Extended Data

## Supplementary Material

Video S1

Video S2

Video S4

Video S3

Video S5

Video S6

Video S7

Video S8

Video S9

Video S11

Supplementary Video legends

Table 1

Table S2

Video S10

## Data Availability

NCBI accession numbers for phages used in this study can be found in Supplementary Table 2; sequences and additional information can be found at phagesdb.org. Genome sequences for phage-resistant strains discovered and used for this study can be found at NCBI BioProject PRJNA862910. Unprocessed imaging data described in this work cannot be deposited in a public repository due to file size limitations. To request access, contact the corresponding authors. Additional data supporting the findings in the current study are available from the corresponding authors upon reasonable request. All biological materials described in this study are available from G.F.H. at gfh@pitt.edu on reasonable request.
