# Supplementary material for: Mycobacterial nucleoid-associated protein Lsr2 is required for productive mycobacteriophage infection
Source: Nat Microbiol. Author manuscript; Available in PMC 2023 Apr 2. (PMC10066036; doi:10.1038/s41564-023-01333-x)
Supplement: Supplementary Video legends [file NIHMS1878592-supplement-Supplementary_Video_legends.docx]

**SUPPLEMENTARY Videos:**

**Supplementary Video 1.** **A time-lapse video of a single *M. smegmatis* cell imaged in multiple channels showing the localization of N-QTF incorporated probe (green) and adsorbed, SYTOX Orange-stained Fionnbharth phages (red).** Individual fluorescent phage particles appear as distinct foci on the surface of the bacterium.

**Supplementary Video 2. Time-lapse of WT *M. smegmatis* cells grown and infected with SYTOX Orange-stained Fionnbharth-mCherry reporter phages in a CellASIC microfluidic device**. Cells are continuously labeled with N-QTF to mark sites of cell wall synthesis and infected via a one-hour pulse of SYTOX Orange-labeled phage particles, which appear as distinct foci on the surfaces of bacteria. The fluorescent protein mCherry is expressed intracellularly from the phage chromosome 1 hour after phage adsorption and manifests as cytoplasmic red signal.

**Supplementary Video 3. Time-lapse of Δ*lsr2* *M. smegmatis* cells grown and infected with SYTOX Orange-stained Fionnbharth-mCherry reporter phages in a CellASIC microfluidic device**. Cells are continuously labeled with N-QTF to mark sites of cell wall synthesis and infected via a one-hour pulse of SYTOX Orange-labeled phage particles, which appear as distinct foci on the surfaces of bacteria. The fluorescent protein mCherry is expressed intracellularly from the phage chromosome 1 hour after phage adsorption and manifests as cytoplasmic red signal.

**Supplementary Video 4.** Time-lapse of Δ*lsr2* *M. smegmatis* cells grown and infected with SYTOX Orange-stained BPs phage particles in a CellASIC microfluidic device. Cells are continuously labeled with N-QTF to mark sites of cell wall synthesis and infected via a one-hour pulse of SYTOX Orange-labeled phage particles, which appear as distinct foci on the surfaces of bacteria.

**Supplementary Video 5.** Time-lapse of WT *M. smegmatis* cells grown and infected with SYTOX Orange-stained BPs phage particles in a CellASIC microfluidic device. Cells are continuously labeled with N-QTF to mark sites of cell wall synthesis and infected via a one-hour pulse of SYTOX Orange-labeled phage particles, which appear as distinct foci on the surfaces of bacteria.

**Supplementary Video 6.** Time-lapse imaging of WT (left) or Δ*lsr2* (right) *M. smegmatis* cells mixed at a ratio of 1:1000 with “seeder” cells infected with the Fionnbharth-mCherry reporter phage and imaged on opposite sides of a 2% agarose 7H9 pad with a 20x objective.

**Supplementary Video 7.** Time-lapse imaging of MalI-mNeonGreen *M. smegmatis* cells grown in a CellAsic microfluidic device and exposed to a short pulse of highly diluted SYTOX Orange-stained Fionnbharth-MalO phage. A single phage binding event (red focus) is followed by the formation of a proximal MalI-mNeonGreen focus (green), consistent with phage ejection and recruitment of cytoplasmic MalI-mNeonGreen protein to MalO sites on the infecting phage chromosome. Over the course of infection, the single green focus multiplies into many foci that spread out across the interior of the cell and then organize regionally into multiple phage replication domains followed by cell lysis and release of phage particles.

**Supplementary Video 8.** Time-lapse imaging of MalI-mNeonGreen *M. smegmatis* cells grown in a CellAsic microfluidic device and exposed to a short pulse of concentrated SYTOX Orange-stained Fionnbharth-MalO phage, which appear as distinct foci on the surfaces of bacteria.

**Supplementary Video 9.** Time-lapse imaging of ∆*lsr2* MalI-mNeonGreen *M. smegmatis* cells grown in a CellAsic microfluidic device and exposed to a short pulse of concentrated SYTOX Orange-stained Fionnbharth-MalO phage, which appear as distinct foci on the surfaces of bacteria.

**Supplementary Video 10.** Time-lapse imaging of Lsr2-Dendra2 *M. smegmatis* cells that contain Lsr2 protein tagged at the native locus with the fluorescent protein Dendra2, grown in a CellAsic microfluidic device and exposed to a short pulse of concentrated SYTOX Orange-stained Fionnbharth phage, which appear as distinct foci on the surfaces of bacteria. 1 hour after phage adsorption, Lsr2-Dendra2 foci near the host DNA replication machinery rapidly relocalize to zones of phage replication.

**Supplementary Video 11.** Time-lapse imaging of Lsr2-Dendra2 *M. smegmatis* cells that contain Lsr2 protein tagged at the native locus with the fluorescent protein Dendra2, grown in a CellAsic microfluidic device and exposed to a short pulse of concentrated SYTOX Orange-stained BPs phage, which appear as distinct foci on the surfaces of bacteria. After phage infection, Lsr2-Dendra2 foci appear to remain associated with the host DNA replication machinery.
