# Supplementary material for: Mycobacterial nucleoid-associated protein Lsr2 is required for productive mycobacteriophage infection
Source: Nat Microbiol. 2023 Feb 23;8(4):695–710. doi: 10.1038/s41564-023-01333-x (PMC10066036; doi:10.1038/s41564-023-01333-x)
Supplement: Supplementary file 1 [file 41564_2023_1333_MOESM1_ESM.pdf]

nature microbiology

Article https://doi.org/10.1038/s41564-023-01333-x

Mycobacterial nucleoid-associated protein Lsr2 is required for productive mycobacteriophage infection

In the format provided by the authors and unedited

Table S1. Primers and gBlocks used in this study

Name	Sequence (5' to 3')	Description
mal-Fionn_fwd	tgcttggtaggagtaccggcTCTGCTCGAGGAATTCTC	add homology to mal array for Fionnbharth upst *43*
mal-Fionn-rev	ccataaatctggactatgttGTAGCCATGCAGATGACC	add homology to mal array for Fionnbharth downst *47*
mal_amp_fwd	TCTGCTCGAGGAATTCTCCAGATTCTAGTG	amplify mal array F
mal_amp_rev	GTAGCCATGCAGATGACCTACTCCCTGATT	amplify mal array R
Fionn upst 43_Rev #2 (cg2520_1)	cggccggtactcctaccaagCACTACACAG	amplify whole construct after assembly F
Fionn downst 47 F #2 (cg_2520_2)	aacatagtccagatttatGGACAAAGCAACTCG	amplify whole construct after assembly R
Fionn upst_43 gblock	ccattgcggcgacggcggCTGAAAGCGCGGTGAGGGACTTCATCGCTGGCTCCAATCCTTTGCTCGT TGGGTGCAGGCCGGCACTGTACCCGAGATTGAGGCGTTTTTGGGTACATGTTTGGGTACACC GCTACGAGCACGGTGCTTCTGACCTGCGGTTTTACGGTGGATTGCACCGGAATCGGCTGATTA AAAGTTCGTCGCCTCGGCGTGTCGACCAGGCATAACACCAGGTCACGCTACCCCGTAGTGCTG TGTAGTGcttggtaggagtaccggc	gblock with homology to Fionnbharth upstream of gene *43*
Fionn downst_47 gblock	aacatagtccagatttatGGACAAAGCAACTCGATTGACGTGCAGATTCTCCAGATCATCGGGCAAC ACGCTGTCAAGATATCCGTAATGATGTCCACAAACCTGGACACAGTGTTGTACCTTCGTAGGC ATGCCAGCCCCTCAGACCTACGAACTCCGATGGAATCCGGAAGCAATCGCAAAACTGCTGCGC CGCAAAAAGATTCACGATCGAGCCGAGCTGGCCGAGGCAATCGGAATCAGCCGCGCAACCGC CTACAACCTGTTCGGCGCTGACTGGTCGGGCAAAGCGACGACCGTTGTCTTGGCCCAACTCGT CGGGTGCCTCGGAGCCAACCCAGCAACCATCGTCGAAACCGTCAAGGTGTCGAAATGACCGC CGATCAGATTCTCGTGACCCGCGACGAGGCCgctcgaatgctgtcgctg	gblock with homology to Fionnbharth downstream of gene *47*
pLO74LinearizeF	GGATCCAGCTGCAGAATTC	linearize calibrated series plasmids (45) removing mCherry
pLO74LinearizeR	GATTGGCTTTCTCCTTTCTC	linearize calibrated series plasmids (45) removing mCherry
lsr2 gblock	GAGAAAGGAGAAAGCCAATCATGGCAAAGAAAGTGACCGTCACGCTTGTCGATGATTTCGAC GGTGAGGCCACGGCCGACGAAACGGTGGAATTCGGCCTCGACGGTGTGACCTATGAGATCG ACCTTTCCGCAAAAAACGCCGCGAAGTTGCGCAACGATCTGAAGCAGTGGGTCGAGGCGGGC CGTCGCGTCGGCGGACGTAAACGCGGTCGCGCCgcgaccaccacgacccgtggccgcggtgccatcgacc gtgagcagagcgctgcgatccgcgaatgggcccgccgcaacggacacaatgtgtcgacccgcggccggattcctgccgac gtgatcgacgcattccacgcggcaacttagggatccagctgcagaattcg	*lsr2* gblock with homology to calibrated series plasmids pLO73, pLO76 (45) to create plasmids pCG52, pCG54
lsr2RARIP gblock	GAGAAAGGAGAAAGCCAATCATGGCAAAGAAAGTGACCGTCACGCTTGTCGATGATTTCGAC GGTGAGGCCACGGCCGACGAAACGGTGGAATTCGGCCTCGACGGTGTGACCTATGAGATCG ACCTTTCCGCAAAAAACGCCGCGAAGTTGCGCAACGATCTGAAGCAGTGGGTCGAGGCGGGC CGTCGCGTCGGCGGACGTAAACGCGGTCGCGCCgcgaccaccacgacccgtggccgcggtgccatcgacc gtgagcagagcgctgcgatccgcgaatgggcccgccgcaacggacacaatgtgtcgacccgcgcccggattcctgccgac gtgatcgacgcattccacgcggcaacttagggatccagctgcagaattcg	*lsr2* gblock G100A with homology to calibrated series plasmids pLO76 (45) to create plasmids pCG67

Table S2. Phages and their Accession numbers used in this study.

Phage Name[1]	Host	Cluster	Subcluster	Temperate?	Accession #	Genome Length(bp)	GC%	lsr2 gene?
Bxb1	Mycobacterium smegmatis mc²155	A	A1	TRUE	AF271693	50550	63.6	No
D29	Mycobacterium smegmatis mc²155	A	A2	TRUE	AF022214	49127	63.5	No
LadyBird	Mycobacterium smegmatis mc²155	A	A2	TRUE	KT588442	53141	63.5	Yes
Fred313	Mycobacterium smegmatis mc²155	A	A3	TRUE	MF373840	50053	64	No
Isca	Mycobacterium sp. GD01	A	A3	TRUE	MN586063	49968	64.2	No
Phrappuccino	Mycobacterium smegmatis mc²155	AA	None	FALSE	MK937592	136361	67.4	No
Muddy	Mycobacterium smegmatis mc²155	AB	None	FALSE	KF024728	48228	58.8	No
Dori	Mycobacterium smegmatis mc²155	AD	None	None	JN698995	64613	66	No
DuchessDung	Mycobacterium smegmatis mc²155	B	B1	FALSE	MH051251	68235	66.4	No
Cjw1	Mycobacterium smegmatis mc²155	E	None	TRUE	AY129331	75931	63.7	Yes
BPs	Mycobacterium smegmatis mc²155	G	G1	TRUE	EU568876	41901	66.6	No
Courthouse	Mycobacterium smegmatis mc²155	J	None	TRUE	JN698997	110569	60.9	Yes
Adephagia	Mycobacterium smegmatis mc²155	K	K1	TRUE	JF704105	59646	66.6	No
ZoeJ	Mycobacterium smegmatis mc²155	K	K2	TRUE	KJ510412	57315	68.5	No
ShedlockHolmes	Mycobacterium smegmatis mc²155	K	K3	TRUE	KR080206	61081	67.3	No
Fionnbharth	Mycobacterium smegmatis mc²155	K	K4	TRUE	JN831653	58076	68	No
LeBron	Mycobacterium smegmatis mc²155	L	L1	TRUE	HM152763	73453	58.8	No
Gabriela	Mycobacterium smegmatis mc²155	L	L2	TRUE	MN703406	75383	58.8	Yes
Nanosmite	Mycobacterium smegmatis mc²155	M	M3	TRUE	MW578836	82676	61.7	Yes
Charlie	Mycobacterium smegmatis mc²155	N	None	TRUE	JN256079	43036	66.3	No
Kumao	Mycobacterium smegmatis mc²155	Singleton	None	None	MG009575	70373	62.1	Yes
LilSpotty	Mycobacterium smegmatis mc²155	Singleton	None	None	MK977707	49798	64.7	No
Sparky	Mycobacterium smegmatis mc²155	Singleton	None	None	KM083128	63334	65.2	No
RonRayGun	Mycobacterium smegmatis mc²155	T	None	TRUE	KM591905	42596	66.1	No
Gaia	Mycobacterium smegmatis mc²155	X	None	TRUE	KJ567043	90460	56.8	Yes
Rem711	Mycobacterium smegmatis mc²155	Z	None	TRUE	MG770216	50832	66.2	No

[1]Phages names and other information as available at https://phagesdb.org
